# Electrostatic Forces Mediate the Specificity of RHO GTPase-GDI Interactions

**DOI:** 10.3390/ijms222212493

**Published:** 2021-11-19

**Authors:** Niloufar Mosaddeghzadeh, Neda S. Kazemein Jasemi, Jisca Majolée, Si-Cai Zhang, Peter L. Hordijk, Radovan Dvorsky, Mohammad Reza Ahmadian

**Affiliations:** 1 Institute of Biochemistry and Molecular Biology II, Medical Faculty and University Hospital Düsseldorf, Heinrich Heine University Düsseldorf, 40225 Düsseldorf, Germany; mosaddeg@uni-duesseldorf.de (N.M.); neda.jasemi@hhu.de (N.S.K.J.); sicaichang@gmail.com (S.-C.Z.); radovan.dvorsky@gmail.com (R.D.); 2 Department of Physiology, Amsterdam UMC, Location VUmc, De Boelelaan, 1108 Amsterdam, The Netherlands; j.majolee@amsterdamumc.nl (J.M.); p.hordijk@amsterdamumc.nl (P.L.H.); 3 Institute of Future Agriculture, Northwest A&F University, Xianyang 712100, China

**Keywords:** CDC42, electrostatic steering, G domain, hypervariable region, geranylgeranyl, guanine nucleotide dissociation inhibitors, liposomes, membrane extraction, polybasic motif, RAC1, RAC2, RHOA, RHOGDI

## Abstract

Three decades of research have documented the spatiotemporal dynamics of RHO family GTPase membrane extraction regulated by guanine nucleotide dissociation inhibitors (GDIs), but the interplay of the kinetic mechanism and structural specificity of these interactions is as yet unresolved. To address this, we reconstituted the GDI-controlled spatial segregation of geranylgeranylated RHO protein RAC1 in vitro. Various biochemical and biophysical measurements provided unprecedented mechanistic details for GDI function with respect to RHO protein dynamics. We determined that membrane extraction of RHO GTPases by GDI occurs via a 3-step mechanism: (1) GDI non-specifically associates with the switch regions of the RHO GTPases; (2) an electrostatic switch determines the interaction specificity between the C-terminal polybasic region of RHO GTPases and two distinct negatively-charged clusters of GDI1; (3) a non-specific displacement of geranylgeranyl moiety from the membrane sequesters it into a hydrophobic cleft, effectively shielding it from the aqueous milieu. This study substantially extends the model for the mechanism of GDI-regulated RHO GTPase extraction from the membrane, and could have implications for clinical studies and drug development.

## 1. Introduction

The RHO family GTPases, most prominently RAC1, CDC42, and RHOA, share two common functional characteristics, membrane anchorage and an on/off switch cycle [1]. They typically contain a conserved GDP/GTP binding domain, called G domain, and a C-terminal hypervariable region (HVR) ending with a consensus sequence known as CAAX (C is cysteine, A is any aliphatic amino acid, and X is any amino acid). Subcellular localization, which is critical for the biological activity of RHO GTPases, is achieved by a series of posttranslational modifications at the cysteine residue in the CAAX motif, including isoprenylation (geranylgeranyl or farnesyl), endoproteolysis and carboxyl methylation [2]. Membrane-associated RHO GTPases act then, with some exceptions [3], as molecular switches by cycling between an inactive GDP-bound state and an active GTP-bound state. This cycle underlies two critical intrinsic functions, GDP-GTP exchange and GTP hydrolysis, which induce structural rearrangements of two regions of the protein, called switch I and switch II [3] (encompassing amino acids 29–42 and 62–68, respectively) and is controlled by two classes of regulatory proteins, guanine nucleotide exchange factors (GEFs) and GTPase activating proteins (GAPs) [4]. RHO GTPases act as dynamic switches in many developmental and cellular contexts [5] by selectively binding to and activating structurally and functionally diverse effectors. This class of proteins activates a wide variety of downstream signaling cascades [6,7,8,9], thereby regulating many important physiological and pathophysiological processes in eukaryotic cells [10,11,12].

The spatial and temporal activation of RHO GTPases inside a cell is fundamental, for example, to the regulation of local movements and cell-cell contacts that are required for morphogenesis [12]. They are commonly found to cycle between two pools, a membrane-associated and a cytosolic pool. Given the fact that membrane attachment is a prerequisite for the signaling roles of this protein family, it is clear that reversible membrane translocation offers cells a means to regulate the location of the activation event. However, there is a serious handicap to such physical cycling for RHO GTPases. The highly hydrophobic geranylgeranyl (GG) moiety of RHO proteins renders them energetically unfavorable to partition into the cytosol as individual monomers. Post-translationally modified RHO proteins can only detach from membranes if they are assisted by RHO-specific guanine nucleotide dissociation inhibitors (GDIs), that shield the bulky lipid moieties from the aqueous environment of the cytosol [4,13].

In contrast to the tremendous number of the other regulatory proteins of the RHO family (74 GEFs and 66 GAPs) [14,15], only three GDIs exist in the human genome [16]. The GDI family includes the ubiquitously expressed GDI1 (or GDIα) [17], GDI2 (GDIβ, LY-GDI or D4-GDI), which is mainly found in hematopoietic tissue, particularly in B- and T-lymphocytes [18], and GDI3 (or GDIγ) that is preferentially expressed in brain, pancreas, lung, kidney, and testis [19]. Unlike the other two GDIs, GDI3 contains an N-terminal extension that confers anchorage into the membranes of Golgi vesicles [20]. As GDI3 is very similar to GDI1, it can form a complex with all the GDI1 targets [21]. GDI1 and GDI2 contain at their very N-terminus a large number of acidic residues which have been proposed to be essential for their function in the cell [22]. In addition to their physiological expression, GDIs are expressed in several human cancers, including breast, liver, ovarian, pancreatic and myeloid leukemia [13,18,23,24]. Changes in GDI expression levels have shown pro-tumorigenic or anti-tumorigenic effects, that are cell type- and tissue-dependent. One reason for these opposite effects is most probably due to our lack of understanding of the basic mechanism of GDI function and their binding specificities to the different RHO proteins.

Understanding the mechanisms by which signaling events are localized and the physiological consequences of spatial restriction are exerted, is one of the major challenges in cell biology. Comprehensive studies in the last three decades have provided insight into the structure and function of these regulators acting as a shuttle for the RHO GTPases [13,25,26,27]. The shuttling process, which considerably differs from the KRAS4B^Far^-PDEδ [28,29,30,31], involves the extraction of RHO GTPases from donor membranes, formation of cytosolic GDI-RHO GTPase complexes and delivery of RHO GTPases to the target membranes [13,27]. Accordingly, it has been proposed that GDI regulates the isoprenylation process in the cell [32]. GDI is known to extract RHO GTPases from the membrane, maintain them in an inactivated state, and protect them from both degradation and unspecific activation by RHO-specific GEFs [13,33,34]. Structural studies by different groups have revealed two sites of interaction between GDI and RHO GTPases [35,36,37,38,39]. First, an N-terminal region of the GDIs binds to the switch regions of RHO GTPases leading to the inhibition of both GDP dissociation and GTP hydrolysis. This step involves the Thr35 from the switch I region which would contact the Mg^2+^ ion and has an indispensable role in nucleotide binding of RHO GTPases. The Asp45 residue from the N-terminal region of the GDIs forms a hydrogen bond with this Thr35 residue of switch I region and increases the affinity for the bound GDP nucleotide, that may explain the preference of GDIs for GDP bound form of RHO proteins [37,38]. The first step positions the GDIs hydrophobic pocket toward the membrane surface. In the second step, the geranylgeranyl moiety of RHO GTPases moves out of the membrane and inserts into the hydrophobic pocket of the GDI molecule after which the formed complex moves to the cytosol [36,38]. The membrane release of CDC42•GDI1 complex occurs at a similar rate as the release of CDC42 alone, with the major effect of GDI1 being to impede the re-association of CDC42 with membranes [40]. Moreover, it has been reported that GDI1 binds the isoprenylated RAC1 and RHOA proteins with extremely high binding affinities 0.4 and 0.005 nanomolar affinity, respectively, as compared to non-isoprenylated proteins [41,42]. Although these studies have clearly demonstrated how GDIs interact with and serve as negative regulators of RHO GTPases, yet the basic mechanisms of how they pull the isoprenoid moiety from the membrane remain elusive.

In this study, we investigated the interaction between the three GDI proteins and various members of the RHO GTPase family using structure-function evaluation as well as kinetic and equilibrium measurements. We found clear specificity for the RHO GTPase-GDI interactions, and substantially extended the existing model for binding and membrane extraction of RHO GTPases by GDI1. We found, that: (1) the geranylgeranyl moiety is dispensable for RAC1-GDI1 interaction, (2) seven out twelve RHO family GTPases did not interact with GDI1, (3) a conserved G domain is not rate-limiting for the GDI1 binding, (4) RAC1 polybasic motif dictates GDI1 binding, (5) electrostatic pincer residues of GDI1 grasp RAC1 HVR, and (5) GDI1 buckles RAC1 into its site.

## 2. Results and Discussion

### 2.1. Geranylgeranyl Moiety Is Dispensable for RAC1-GDI1 Interaction

To understand the impact of the isoprenyl moiety of RAC1 on GDI1 binding, we compared the biochemical properties of geranylgeranylated RAC1 (RAC1^GG^) from insect cells and non-isoprenylated RAC1 full-length (RAC1^FL^) from *Escherichia coli*. Previous mass spectrometric analysis and liposome sedimentation of intact RAC1^GG^, compared to RAC1^FL^, have revealed a fully modified RAC1^GG^ by geranylgeranylation [34]. Unless otherwise stated, the unmodified RAC1 purified from *E. coli* is designated as RAC1^FL^, the modified RAC1 purified from insect cells as RAC1^GG^, and the unmutated (wild-type) RAC1 as RAC1^WT^ in cell-based experiments.

**Figure 1:**
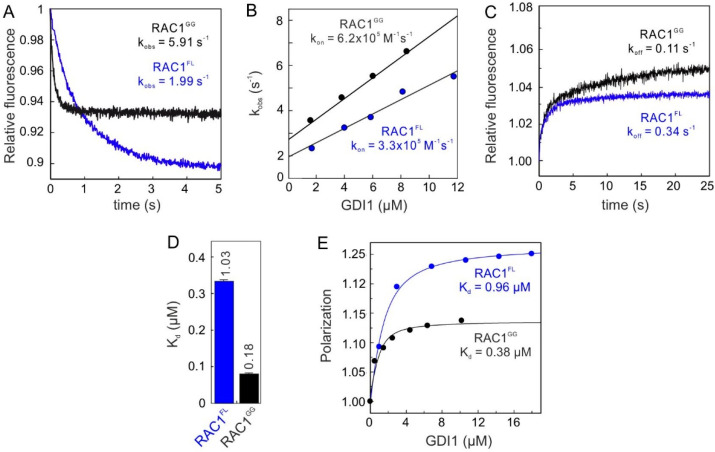
RAC1^GG^ and RAC1^FL^ bind with a similar affinity to GDI1. (**A**) Association of GDI1 (4 µM) with RAC1^GG^ and non-isoprenylated RAC1^FL^ (0.2 µM, respectively). (**B**–**D**) Quantitative measurements of GDI1 interaction with RAC1^GG^ and RAC1^FL^ led to the calculation of the individual binding constants, association rate constant or k_on_ (**B**), dissociation rate constant or k_off_ (**C**), and dissociation constant or K_d_ directly from the k_off_/k_on_ ratio (**D**). (**E**) Titration of increasing GDI1 concentrations to RAC1^GG^ and non-isoprenylated RAC1^FL^ (0.2 µM, respectively), using fluorescence polarization, resulted in the determination of the equilibrium K_d_ values.

We determined the GDI association rates with both RAC1^GG^ and RAC1^FL^ using a stopped-flow fluorometric assay. Figure 1A shows a rapid decrease in fluorescence after mixing GDI1 with the RAC1 proteins, which is directly related to the association reaction between the RAC1-GDI1 pairs. Observed rate constants (k_obs_) obtained by a single exponential fitting increased linearly as a function of the GDI1 concentrations (Figure 1B), and yielded similar association rate constants (k_on_) for both RAC1^GG^ and RAC1^FL^. The dissociation of the GDI1 from mdGDP-bound RAC1 proteins was measured in a displacement experiment. Observed single exponential fluorescence increase yielded respective dissociation rate constants (k_off_), which differ only 3-fold (Figure 1C). Notably, the RAC1^GG^-GDI1 interaction showed a biphasic behavior (double exponential kinetics); particularly for the off-rate, an initial rapid increase in fluorescence was followed by a slow plateau phase, which can be attributed to the GG moiety of RAC1^GG^. Calculated dissociation constants (K_d_) from the ratio of the k_off_ and k_on_ values (Figure 1D) unexpectedly revealed only a 7-fold higher affinity for RAC1^GG^ vs. RAC1^FL^.

Instead of the simple exponential decrease seen with GAP, there is an initial rapid increase in fluorescence followed by a decrease at a rate similar to that seen at high GAP concentrations. The first phase of the reaction is concentration-dependent, whereas the second is less obviously so. This suggests that an initial association between the proteins leads to an increase in fluorescence, which then decreases on mant-GTP hydrolysis (and consequent dissociation of the proteins) to a level below that of the starting level, in agreement with the observation that the fluorescence of Ras‚mant-GDP is lower than that of Ras‚mant-GTP. This interpretation means that the mechanism cannot be treated as a very rapid initial equilibration followed by a relatively slow cleavage step, which is the underlying assumption in the analysis of the data obtained with GAP.

This result clearly contradicts existing models which have suggested that the isoprenyl moiety of the RHO GTPases contributes to orders of magnitude higher binding affinity for GDI as compared to unmodified RHO GTPases [41,42]. The studies have determined K_d_ values of 0.4 nM and 5 pM for the interaction of GDI1 with prenylated RAC1 and RHOA, respectively, as compared to the 180 nanomolar K_d_ value determined in our study. The reason for the extraordinarily large differences between the binding affinities obviously lies in the proteins and the type of fluorescence reporter groups used in each case. On the one hand, we and Tnimov et al. applied native GDI1 while Newcombe et al. [41] used a coumarin-labeled GDI1 at position cysteine 79, which is actually buried and located in the back side of the C-terminal geranylgeranyl binding domain (GGBD); this modification at this position can drastically alter the confirmation and consequently its biochemical properties of GDI1. On the other hand, native RAC1^GG^ purified from insect cells was used by Newcombe et al. as well as in this study, Tnimov et al. [42] used a cell-free modification approach of RHOA^FL^ and purified geranylgeranyl transferase I and nitrobenzoxadiazole (NBD)-labeled geranyl-pyrophosphate [1]. In this way, the nature of the isoprenyl moiety and the absence of further posttranslational modifications by specific proteolytic removal of the terminal three residues, and carboxylmethylation of prenylted cysteine residue, are largely different from native RHOA^GG^.

Kinetic and equilibrium data revealed that the RAC1-GDI1 interaction is not markedly dependent on the presence of the geranylgeranyl moiety. The binding affinities determined for the RAC1-GDI1 interaction by three different methods indicate that GDI1 binds to non-isoprenylated RAC1 as efficiently as it does to isoprenylated RAC1; RAC1^GG^ exhibited only 2.5 to 4.6-fold lower K_d_ values compared to RAC1^FL^. Evidently, these data clearly challenge the current regulatory model that the isoprenyl moiety at the C-terminus of RHO GTPases contributes to several orders higher binding affinity of GDI1.

The data presented in this study on similar binding affinity of GDI1 for RAC1^GG^ and RAC1^FL^ is not surprising for two reasons: (1) It is evident that GDI1 recognizes and binds regions of RAC1 other than the geranylgeranyl moiety in the cell because the latter is inserted into the lipid bilayer and anchors the RAC1 to cellular membranes. Thus, binding of the geranylgeranyl moiety by GDI1 cannot be rate-limiting. (2) GDI1 may not get into a situation where it competes for prenylated and nonprenylated RAC1 simply because nonprenylated RHO GTPases, such as RAC1, may not exist in cells; this has not been reported to our knowledge. Our data can provide an indication of the interaction specificity of GDIs for RHO GTPases. Therefore, we thoroughly investigated the specificity and the structure-function relationships of the three GDIs and various RHO GTPases.

### 2.2. Conserved G Domain Is Not Rate-Limiting for the GDI1 Binding

**Figure 2:**
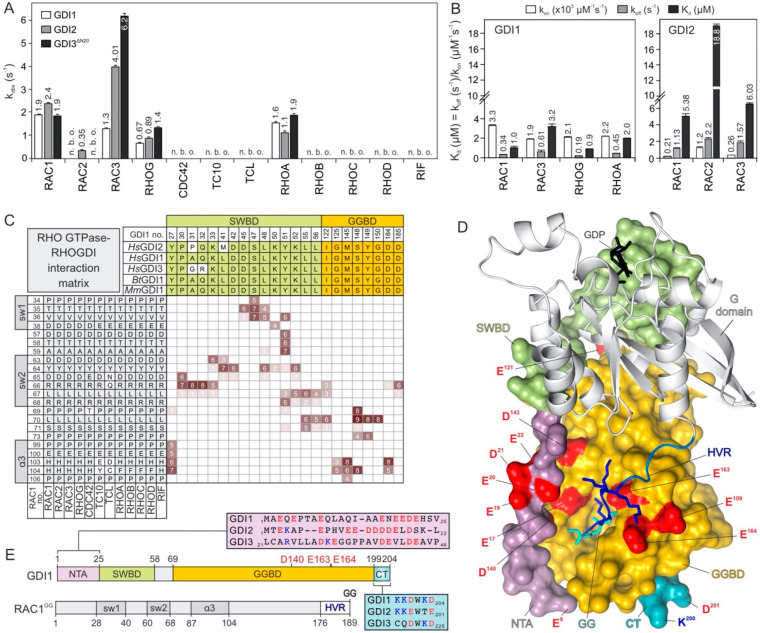
Biochemical and structural view into the RHO GTPase-GDI interactions. (**A**) Kinetics of association of 2 µM GDI proteins (GDI1, GDI2 and GDI3ΔN20) with 0.2 µM mdGDP-bound RHO GTPases (twelve different proteins) was only monitored for RAC1, RAC3, RHOG and RHOA using stopped-flow fluorimetry. GDI2 but not GDI1 and GDI3 associated with RAC2. No binding was observed (n. b. o.) for the other GTPases. Obtained k_obs_ values are the average of four to six independent fluorescence measurements, consisting of 1000 data points each (mean ± S.D.). Kinetic data are shown in Figure S2. (**B**) Individual rate constants were determined, under the same conditions as shown in Figure 1A–C, for the interaction of GDI1 with RAC1, RAC3, RHOG and RHOA, and GDI2 with RAC1, RAC2 and RAC3, respectively. Kinetic data were derived from the average of four to six independent measurements (mean ±S.D.). Kinetic data are shown in Figure S3. (**C**) An interaction matrix of the GDI proteins with twelve RHO family GTPases is generated to determine the frequency of contacts in respective structures. (see Table S2; for more detail see Figure S4). It comprises the amino acid sequence alignments of the RHO proteins (lower left panel) and the GDIs (upper right panel), respectively. Each element corresponds to a possible interaction of RHO residues (row; RAC numbering) and GDI residues (column; GDI1 numbering). The number of actual contact sites between RHO and GDI proteins (with distances of 4 Å or less) were calculated and are indicated with numbers for matrix elements between 1 and 9. (**D**) A detailed view into the structure (PDB code: 1HH4) of GDP-bound RAC1^GG^ (grey ribbon) in complex with GDI1 (surface representation) revealed that the basic HVR (blue) is sandwiched between a series of acidic residues of GDI1 supplied by NTA (purple) and GGBD (orange). (**E**) Schematic diagrams of the domain organizations of GDI1 and RAC1^GG^ illustrate their detailed boundaries. Amino acid sequence alignments of the N-terminal arm (NTA; 25 amino acids) and the C-terminal six residues of the GDI proteins (boxed) highlight negatively and positively charged residues (red and blue). Colors are the same in (**D**).

The above findings prompted us to unambiguously challenge the paradigm of RAC1 regulation by GDI1. To this end, we investigated the interactions of the three GDI paralogs, GDI1, GDI2 and GDI3, with various non-isoprenylated members of the RHO GTPase family (Table S1; Figure S1). GDI3 was purified as an N-terminal truncated variant lacking the amphipathic helix (amino acids or aa 1-20). Kinetics of association of the GDIs (2 µM) with 0.2 µM mdGDP-bound RHO GTPase was monitored under the same conditions as described above for RAC1. Calculated k_obs_ values for each measurement (Figure S2) were plotted as bar charts in Figure 2A, which clearly show that all three GDIs associated with RAC1, RAC3, RHOG and RHOA under the experimental conditions but apparently not with RAC2, CDC42, TC10, TCL, RHOB, RHOC, RHOD and RIF. GDI2 and GDI3 showed significantly faster association with RAC3 as compared to GDI1. Most remarkably, unlike GDI1 and GDI3, GDI2 was able to bind RAC2. These results clearly indicate for the very first time that GDIs can discriminate between the RHO GTPases by interacting with some but not all the RHO proteins.

To examine binding properties, the respective association rate constants (k_on_) and the dissociation rate constants (k_off_) were determined for the interaction of GDI1 with RAC1, RAC3, RHOG and RHOA, and GDI2 with RAC2 under conditions described above (Figure 1A–D and Figure S3A). All kinetic parameters along with calculated dissociation constants (K_d_) are summarized in Figure 2B. The data are very similar for the GDI1 interaction with RAC1, RAC3, RHOG and RHOA with K_d_ values between 0.9 and 3.2 µM. However, GDI2 interactions exhibited similar rate constants for RAC1 and RAC3, which were significantly different from that of GDI1 (Figure 2B). GDI2 exhibited more than 10-fold faster k_on_ values and up to 4-fold slower k_off_ values. GDI2-RAC2 interaction was characterized by a much slower rate of association as compared to RAC1 and RAC3, resulting in a binding affinity of 18.8 µM under the given experimental conditions (Figure 2B).

The biochemical characterization together with structural studies has shown that the RAC paralogs exhibit different properties concerning ligand– and protein–protein interactions [43]. Whereas RAC1 and RAC3 behave almost identically, RAC2 revealed a 25-fold lower nucleotide affinity because of a decreased nucleotide association rate, a slightly higher PAK1 (p21 activated kinase-1, a downstream effector for RAC1 and CDC42) binding affinity, and a significant increase in GEF-catalyzed nucleotide dissociation. These aberrant properties most likely are the consequence of different conformational flexibilities in the switch I region [44].

To understand this result, we have performed integrated sequence-structure analysis for all available GDI structures in complex with RHO GTPases (Table S2) and identified amino acids of the G domain (aa 1–176) involved in RHO GTPase-GDI interactions shown as an interaction matrix in Figure 2C. It reveals that almost all of them are indentical in different RHO GTPases. GDIs appear to mainly contact RHO GTPases through the switch I and II regions, and the α-helix 3 (shown in grey). The same is true also for the GDIs, which apply identical residues, with a few exceptions, to contact RHO GTPases. A major part of the contacts stems from the N-terminal switch binding domain (SWBD in green) and some from the GGBD (in orange; Figure 2D,E). So, identical contact sites do not explain the observed differences in kinetic measurements and this finding rather suggests a GDI1 modulatory region outside the G domain, namely the HVR.

A comparison of available structures of the RAC1-GDI1 and RAC2-GDI2 complexes revealed a rather high sequence similarity [35,36]. An inspection of full interaction matrix revealed very few amino acid deviations within the RAC G domains (Y/F89) and GDI paralogs (A/P/G31, E/K/R53, A/T/V54; Figure S4). However, Y98 in RAC1 and RAC3 undergoes contacts with H23 and V25 of GDI1 which may not be achieved by F89 in RAC2, that may only contact V25 but not S23. Among the deviations in GDIs, the loop containing A/G31 in GDI1 and GDI3 is in close vicinity of D65/R66 of RAC paralogs, which may, in the case of P31 in RAC2, adopt a different orientation and thereby influence RAC2-GDI2 binding. Residues in SWBD, such as E53 and A54 (GDI1 numbering), appear to be critical for RHO GTPase-GDI interaction. The double mutation of L55/L56 to serines in GDI1 has been shown to drastically decrease its affinity for RAC1 [45].

The interaction matrix showed a conserved interface between RHO GTPases and GDIs, but the missing parts of most structures are, on the one hand, the C-terminal HVR (178–190 aa in RAC1; Figure 2E) [46], and on the other hand, the N-terminal of the GDIs (1–25 aa in GDI1; Figure 2D) [22]. The crystal structure of the RAC1^GG^•GDP•GDI1 complex (PDB code: 1HH4) has remarkably provided the first evidence for the existence of a network of electrostatic interactions between these otherwise highly flexible regions [35]. Accordingly, GDI1 obviously supplies two sets of negatively charged residues to grasp the polybasic motif of RAC1 HVR. These are E109, D140, E163 and E164 of GDI1 GGBD across from E17, E19, E20, D21 and E22 at the flexible NTA of GDI1 (Figure 2E). Thus, it seems that GDI1 applies an electrostatic pincer towards the polybasic motif of RAC1 and extracts it from the membrane. This mechanistic model was next investigated in-depth.

### 2.3. RAC1 Polybasic Motif Dictates GDI1 Binding

**Figure 3:**
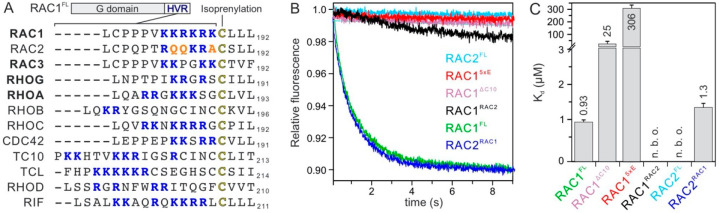
RAC1 HVR generates a selective and high affinity interaction toward GDI1. (**A**) A sequence alignment of hypervariable region (HVR) of RHO GTPases shows significant differences in the frequency of the basic residues (blue). GDI-binding proteins are shown in bold. Critical amino acid deviations in RAC2 are shown in orange. The isoprenylation site (cysteine 189 in RAC1) is highlighted in bold. (**B**) Kinetics of GDI1 association were measured by mixing RAC1 and RAC2 variants (0.2 µM, respectively) with 2 µM GDI1. (**C**) K_d_ values for the RHO GTPase-GDI1 interaction were determined by titrating RAC1 and RAC2 variants (0.2 µM, respectively) with increasing concentrations of GDI1 using fluorescence polarization (see Figure S5 for more details).

A sequence analysis of HVRs of GDI1 associating RHO GTPases (‘binders’) versus those with no observed GDI1 association (‘non-binders’) showed clear differences in both numbers and relative positions of positively charged residues (Figure 3A). To examine the impact of HVR on the RHO GTPase-GDI1 interaction, we measured the kinetics of GDI1 association with different HVR variants of RAC1 and RAC2. Remarkably, a loss of RAC1 association was observed with a C-terminal truncated variant lacking HVR-CAAX (RAC1^ΔC10^) as well as KRKRK-to-EEEEE (RAC1^5xE^; charge-reversal variant) and KRKRK-to-QQKRA (RAC1-to-RAC2 or RAC1^RAC2^ variant). In contrast, a gain of GDI association with RAC2 was observed with QQKRA-to-KRKRK (RAC2-to-RAC1 or RAC2^RAC1^ variant; Figure 3B). These findings were verified by fluorescence polarization experiments (Figure S3A) and obtained data summarized in Figure 3C revealed that (1) RAC1^ΔC10^ yet bound GDI1 with a 26-fold lower affinity as compared to RAC1, (2) RAC1^5xE^, binding to GDI1 was yet observed with a very low affinity while this was not possible for RAC1^RAC2^, and (3) RAC2^RAC1^ did, in contrast to RAC2, bind GDI1 with an almost similar affinity as determined for RAC1. Taken together aided HVR alteration can completely abolish GDI1 association with RAC1 and revert GDI1 association with RAC2.

The results clearly demonstrate the critical role of the polybasic motif of RAC1 in determining GDI1 binding. It seems that both an increase of overall positive charge in HVR of RAC2 and the distance of the basic residues from the geranylgeranyl site strongly reinforce GDI1 binding affinity. We hypothesize that the GDI1 selectively binds RAC1 polybasic motif to pull the GG moiety from the plasma membrane and direct it into the hydrophobic cavity of its GGBD.

The relative position and the order of the basic residues in HVR seem to contribute to the formation of an electrostatic network (Figure 2E) that may significantly stabilize GDI1 interaction with, for example, RAC1 and RAC3 but not RAC2. Synthetic peptides containing the polybasic motifs of RAC1 (aa 178–188), but not RAC2 (178–188), have been shown to inhibit NADPH oxidase activity in a RAC1-dependent system, and interfere with the translocation of RAC1 proteins to the plasma membrane [47]. While the geranylgeranyl moiety mediates membrane anchorage, the polybasic motif of RAC1 interacts with plasma membrane phosphoinositides and stabilizes its proper orientation [48].

Considering the amino acid sequence identity of the G domain on the one hand (Figure 2C), and the sequence similarities among the hypervariable regions on the other (Figure 3A), it is striking that seven out twelve RHO family GTPases do not interact with GDI1 (Figure 2A). For example, the HVRs of RHOA versus RHOC and RAC3 versus CDC42 look very similar, and yet GDI1 binds one but not the other, under the same experimental condition in this study. An in-depth analysis of the RAC1 and RAC2 variants revealed that the HVR polybasic motif dictates GDI1 binding (Figure 3B,C). The number of positively charged residues appears not to be a binding determining factor since the HVR polybasic motif of the nonbinder RHOC has a higher positive net charge as compared to the binder RHOA (Figure 3A). This is also true if comparing the binder RAC3 with the nonbinder CDC42, which exhibits a much larger amino acid variability with their HVRs. Thus, we assume at this stage that, not the number of positive charges but rather the position of the basic residues relative to the C-terminal cysteine determines the bilateral binding selectivity of the HVR polybasic motif by the negatively charged residues of both GGBD and NTA (Figure 2D,E). This may be the reason for TC10 and RHOD not to interact with GDI1 related to the distance of the polybasic motif to the C-terminal Cysteine (Figure 3A). Notably, Gosser et al. has reported a binding affinity of 1.6 nM between unmodified CDC42 and GDI1, which significantly impaired upon N-terminal deletions of GDI [49]. This value is three orders of magnitude lower than the K_d_ values we have obtained from kinetic and equilibrium measurements (Figure 1). Gosser et al. have used in addition to mGDP-bound CDC42 also a fluorescein-conjugated GDI at position cysteine 79, which is actually buried and located in the back side of the GGBD; this modification at this position can drastically alter the confirmation and consequently int biochemical properties of GDI1. Moreover, residues next to the positively charged residues within the HVR seem to play a role, too. Glutamines in RAC2 and RIF seem to be deleterious for the interaction with GDI1. Glutamate 181 in CDC42 may exert electrostatic repulsive effects on the GDI binding (Figure 3A). Serine 185 is a phosphorylation site on CDC42, regulating its translocation to the cytosol by favoring its interaction with GDI1 [50]. Future studies will shed light on these issues.

### 2.4. Electrostatic Pincer Residues of GDI1 Grasp RAC1 HVR

**Figure 4:**
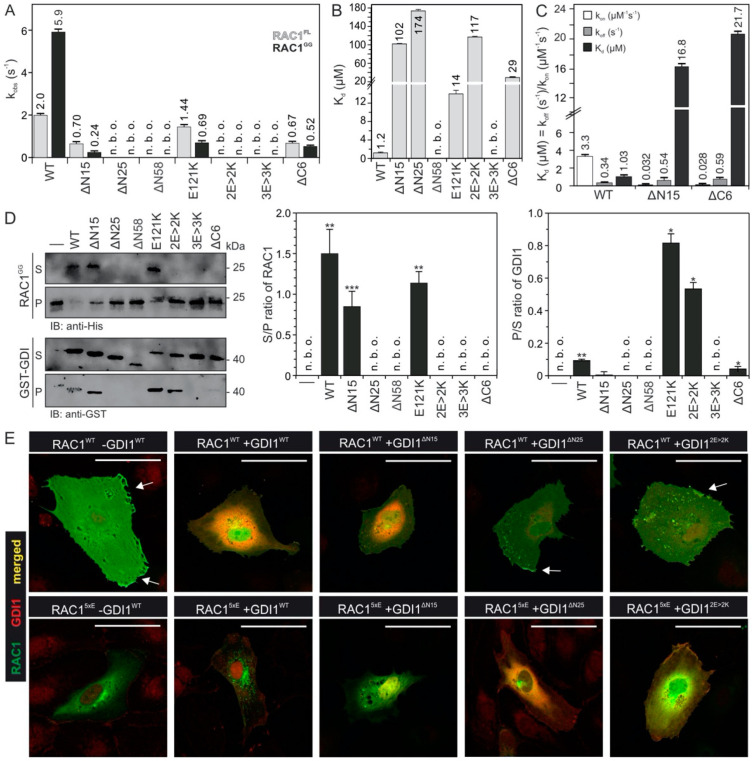
RHOGDI1 grasps basic HVR of RAC1 with multiple negatively charged residues. (**A**) Kinetics of association of 2 µM GDI1 variants with 0.2 µM mdGDP-bound RAC1 was monitored using stopped-flow fluorimetry. The obtained data are the average of four to six independent measurements (mean ± S.D.). Kinetic data are shown in Figure S6. (**B**) K_d_ values for the interaction between the GDI1 variants and mdGDP-bound RAC1 were determined by fluorescence polarization (See Figure S7 for more details). (**C**) Individual rate constants were determined, under the same conditions as shown in Figure 1A–C, for the interaction of RAC1 with GDI1 WT, ΔN15 and ΔC6, respectively. Kinetic data were derived from the average of four to six independent measurements (mean ± S.D.). (**D**) GDI1 variants are, in contrast to GDI1^WT^, impaired in extracting RAC1^GG^ from PIP-enriched synthetic liposomes. The graphs represent densitometric analysis of three independent liposome sedimentation experiments (see Figure S8). Data are expressed as the mean of triplicate experiments ± standard deviation (unpaired t-test, * *p* < 0.05, ** *p* < 0.01, and *** *p* < 0.001). (**E**) FLAG-GDI1 variants were not able to extract YFP-RAC1 from the plasma membrane of HUVECs. All individual images are illustrated in Figure S9. Scale bar represents 50 µm. Arrow point to colocalization of RAC1 and GDI1 at the membrane ruffles.

GDI1 function appears to be driven and controlled by electrostatic forces, that attract the polybasic motif of RAC1. To examine this mechanism, we generated different deletion and charge reversal variants of GDI1 and measured both their binding capabilities to RAC1^GG^ and RAC1^FL^, as well as their functional properties to displace RAC1^GG^ from PIP-enriched synthetic liposomes. GDI1^E121K^, which apparently does not contact RAC1 HVR but the switch 2 region (Figure 2E), was used as a control. Kinetic analysis showed that most GDI variants are disabled in associating with RAC1 (Figure 4A). Substitutions of D140, E163 and E164 for lysines or deletion of the N-terminal and very C-terminal amino acids significantly impaired GDI1 binding to isoprenylated and non-isoprenylated RAC1, as compared to GDI1^WT^. The most drastic effects were observed with 25 and 58 amino acids deleted at the N-terminus (∆N25 and ∆N58) on the one hand, and double (E163K and E164K or 2E > 2K) and triple (E140K, E163K and E164K or 3E > 3K) mutations, on the other, which did not bind to the RAC proteins under the experimental conditions. Fluorescence polarization measurements verified that most GDI1 variants were yet able to bind RAC1^FL^, but with up to 145-fold lower binding affinities compared to GDI1^WT^ (Figure 4B). RAC1 binding was completely abolished in the case of ∆N58 and 3E > 3K variants. GDI1^ΔN58^ not only lacks the very N-terminal acidic resides, that are integral elements of the electrostatic pincer function, but also the switch binding domain (SWBD), which forms multiple contacts with the RAC1 switch regions (Figure 2C,D). GDI1^3E>3K^ most likely creates intermolecular charge repulsion towards positively changed HVR.

**Figure 5:**
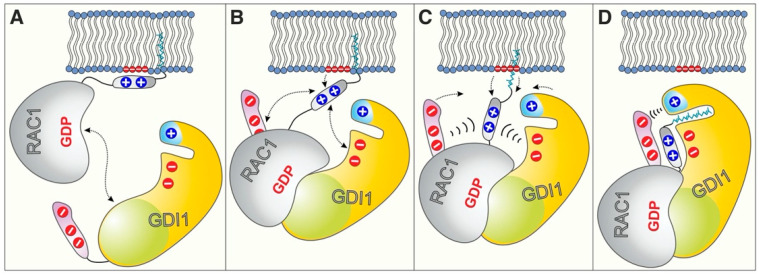
Schematic models of GDI1-regulated RAC1 extraction from the cell membrane. (**A**) GDI1 SWBD (green) recognizes and binds the switch regions of RAC1 to initiate its extraction from the membrane. (**B**) This step is followed by the electrostatic attraction of RAC1 polybasic region through the negatively charged GGBD (orange) and probably also NTA (purple). (**C**) NTA and GGBD are integral elements of the electrostatic pincer function, creating intermolecular charge attraction forces towards positively changed HVR. (**D**) This electrostatic steering mechanism rounds off the GDI1-mediated RAC1 extraction from the membrane by locking the geranylgeranylated C-terminus of RAC1 through the terminal charged regions of GDI1, and safeguarding RAC1^GG^ -bound state of the GDI. Coloring is the same as the coloring of GDI1 and RAC1 structures shown in Figure 2D,E. Positive charges are schematically shown as + and negative electrostatics as −. For more details, see the discussion and conclusions.

To analyze the function of the GDI variants in extracting RAC1^GG^ from the liposomes, we performed a liposome sedimentation assay established previously [34]. Therefore, we mixed PIP-enriched liposomes (200 µm in diameter) with GDP-bound RAC1^GG^ and isolated liposome-bound RAC1^GG^ from the pellet fraction (Figure 4D, first lane) after sedimentation. Next, 1 µM of RAC1^GG^•GDP bound to liposomes was mixed with 2 µM of GDI1 WT and its variants (2 µM, respectively) to measure their ability to displace RAC1^GG^ from the liposomes. Figure 4D shows that GDI1^WT^ quantitatively displaced RAC1^GG^ from the liposomes. In contrast, the majority of the GDI1 variants revealed a significant reduction in their activities, consistently with the kinetic and equilibrium measurements (Figure 4A–C). Particularly, GDI1^ΔN58^ and GDI1^3E>3K^ were completely disabled in binding and extracting RAC1^GG^ from the liposomes, strongly supporting the notion that GDI1 supplies an electrostatic pincer to grasp RAC1 and pull it out from the plasma membrane. Moreover, GDI1^E121K^ and GDI1^2E>2K^ remained partially associated with liposomes and were sedimented in the pellet fraction (Figure 4D). The fact that these GDI1 variants were able to bind to RAC1^GG^ on the liposomes but could not extract it from the liposomes strongly suggests that an electrostatics-guided binding and extraction mechanism is impaired unilaterally. We think that binding of the GDI1 GGBD with the RAC1 HVR takes it away from its membrane association and release additional basic residues on HVR for the interaction with the negatively charged residues of the GDI1 NTA (the so-called electrostatic pincer; Figure 5). Loss of the GGBD-HVR interaction at this step obviously disabled the GDI1 variants (GDI1^E121K^ and GDI1^2E>2K^), that is associated with RAC1^GG^ on the liposomes, to extract RAC1^GG^ from the membrane.

YFP-RAC1 and FLAG-GDI1 variants were ectopically expressed in HUVECs to analyze the molecular basis of their interactions using immunofluorescence (Figure 4E and Figure S9). In the absence of FLAG-GDI1, YFP-RAC1 was both localized in the cytoplasm and at the plasma membrane (arrowhead). When either GDI1^WT^ or GDI^∆N15^ were co-expressed, RAC1 was extracted from the plasma membrane and resided in the cytoplasm. In contrast, GDI^∆N25^ and GDI^2E>2K^ interestingly co-localized with RAC1 at the plasma membrane (arrow head), supporting above data that they still bind RAC1, however were disabled in displacing it from the membrane (Figure 4D). The RAC1 localization pattern in co-expression with GDI^ΔN25^ and GDI^2E>2K^ was similar to the RAC1 localization in the absence of GDI. In contrast, RAC1^5xE^ was exclusively cytosolic both in the absence of GDI1 and in the presence of GDI^ΔN15^, GDI^∆N25^, and GDI^2E>2K^. Co-expression of GDI1 and RAC1^5xE^ seemed to result in the localization of RAC1^5xE^ in the perinuclear structure. Similar to RAC1, co-expression of RAC1^5xE^ without GDI or with GDI^ΔN25^ and GDI^2E>2K^ resulted in a similar RAC1 localization. Furthermore, GDI^ΔN25^ co-localization was stronger when co-expressed with RAC1^5xE^ in comparison to its co-expression with RAC1, which is due to the reduction of repulsion that exists between 25 N-terminal amino acids of GDI and HVR of RAC1^5xE^.

Both GGBD and NTA of GDI1 provide negatively charged residues as the basic building block of the electrostatic pincer of GDI that grips the RAC1 HVR, pulls the geranylgeranyl moiety out of the membrane and push it into the hydrophobic cavity of GGBD (Figure 5). A prerequisite for ensuring the pincer function is the preceding association of GDI1 via SWBD and also GGBD (Figure 2C). GDI1 variants that lacks negatively charged residues in GGBD (GDI^2E>2K^) or NTA (GDI^∆N25^) are able to recognize and bind RAC1^GG^ but are disabled in displacing RAC1^GG^ from the membrane.

The results above are consistent with previous findings and support the concept of an electrostatic pincer mechanism on a subset of RHO family GTPases. An early NMR study has shown that deletion of the highly flexible N-terminal region of GDI1 impairs its ability to extract RAC1 from the plasma membrane in HeLa cells [51]. Mutations of R186 to cysteine in CDC42 HVR has very recently been shown to disrupt its interaction with GDI1 in patients with a novel autoimmune hematological disorder [52]. Thus, electrostatic complementarity between GGBD with the corresponding negative potentials, as shown in this study, and the polybasic region of RAC1, on the one side, and the negative potentials of the N-terminal, rather flexible NTA moving towards the polybasic region from the other side, obviously provide the required forces to facilitate RAC1 displacement from the membrane [35,36,51,53].

### 2.5. GDI1 Buckles RAC1 into Its Site

A closer look into the RAC1^GG^-GDP-GDI1 complex (PDB code: 1HH4) revealed that the very terminal regions of GDI1 may undergo an electrostatic interaction and thus tighten the complex and avoid dissociation. To examine this hypothesis, we functionally analyzed two flanking deletion variants of GDI1 regarding RAC1 binding and membrane extraction. Kinetic analysis showed that the association rate of GDI1^ΔN15^ and GDI1^ΔC6^ were drastically slowed down up to 24-fold, especially for mGDP-bound RAC1^GG^ (Figure 4A). Equilibrium measurements of these terminally deleted variants revealed a massive reduction in the K_d_ values of 440- and 140-fold, respectively, as compared to GDI1^WT^ (Figure 4B). These GDI1 variants also exhibited a reduced activity in RAC1^GG^ extraction from the liposomes (Figure 4D).

Our findings revealed that completing the RAC1-GDI1 interaction is seemingly based on electrostatic steering, selectively dictated by charge-charge interactions. This mechanism is notably represented by almost identical k_off_ rates, and more than 100-fold difference in the k_on_ rate for interaction of GDI wt, ΔN15 and ΔC6 with RAC1 (Figure 4C). The kinetic values disclose, for example, the significance of these negatively charged residues of the very N-terminus of GDI1 (E3, E5 and E9; GDI1 residues 1-8 are not visible in the RAC1-GDI1 structure; Figure 2D,E and Figure 4B) in closing up the RAC1 membrane extraction and shielding the geranylgeranyl moiety. Moreover, GDI1^ΔC6^ exhibited similar attributes to GDI1^ΔN15^ and GDI1^E121K^ in terms of RAC1 binding capability but has a different effect on RAC1 extraction from the liposomes (Figure 4D). This suggests that the CT motif may have additional roles beyond stabilizing the RAC1^GG^-GDI1 complex through an intramolecular NTA-CT interaction.

These data about the roles of the very N-terminal and C-terminal regions of GDI1, which may hold true for GDI2 and GDI3 due to sequence similarities (Figure 2E), strongly suggest that these regions may act as a ‘buckle’ that connects them and safeguards RAC1-bound state of the GDI.

## 3. Conclusions

This study elucidated a distinct and specific mode of GDI function that holds true for only a subset of RHO GTPases. Our data uncovered a latent set of interactions between RAC1 and GDI1, which add additional insight into the multi-step process that facilitates membrane extraction and inhibition of RAC1 activation. We thus hypothesize that GDI1 binds first with its SWBD to the highly conserved switch regions [4] of RAC1^GG^ on the membrane and then by the GGBD the RAC1 HVR associated with the negatively charged phospholipids (Figure 5A,B) [48]. This step may initiate an electrostatic steering mechanism, which, resulting from long-range charge-charge interactions, determines selective recognition of the HVR positive potentials by the GDI1 NTA negative potentials (Figure 5C). This process may generate the required force to pull the geranylgeranyl moiety from the membrane and place it into the GGBD hydrophobic cleft. A last step may be locking RAC1^GG^-GDI1 interaction through the very terminal residues of GDI1 NTA and CT (Figure 5D).

An electrostatic steering mechanism has been previously demonstrated for the interaction between CDC42 and WASP [54,55], and VWF and GPIbα [56]. It results from long-range charge-charge interactions, and dictates selective bimolecular recognition. Notably, electrostatic steering forces control the accelerated association reaction of two molecules, but not the dissociation reaction [54]. This small list of interactions can now be extended to interactions between GDIs and RHO GTPases, such as RAC1.

Our data demonstrated that GDI1 binds essentially similarly to prenylated and nonprenylated RAC1. This is plausible since the geranylgeranyl moiety of RAC1 is inserted into the cellular lipid bilayer and simply prevented from protein interactions. So, what could be the biological implication of the finding that GDI1 binds essentially similarly to RAC1 with and without a geranylgeranyl moiety? The answer probably lies in the specificity of GDIs for RHO GTPases. Our protein interaction and structure-function studies revealed the following insights: (1) The activities of three GDIs do not differ considerably (Figure 2A), pointing to their cell-type specific expression patterns on the one hand and their subcellular localization on the other, providing two GDIs, e.g., GDI1 and GDI3, are expressed at the same time in the same cell. GDI2 showed the highest k_on_ value for RAC2, associating up to 6-fold faster than RAC1 and RAC3. (2) The three GDIs exhibited a clear specificity for the RAC-like proteins, RAC1, RAC2, RAC3 and RHOG, as well as for RHOA, under the experimental conditions used in this study. A systematic analysis of the sequence-structure-function relationships of the RHO GTPase-RHOGDI interaction identified the C-terminal HVR of the RHO GTPases as the key element that ascertains the GDI specificity. (3) Neither GDI1 nor GDI3 but GDI2 exhibited clear selectivity for RAC2, even though this interaction is one of the weakest interactions measured in this study (Figure 2A,B). To explain the RAC2-GDI2 interaction selectivity, which can preferably take place in the hematopoietic system [57,58]. we inspected the RHO GTPase-GDI interaction matrix in detail and found amino acids in GDI2 deviating from those in GDI1 and GDI3, namely Pro28, Leu35, Met38, Asp39, Ala139 and Phe141 (Figure S3). The latter two residues which undergo contact to RHO GTPase HVR may be critical as changes in HVR such as RAC1-to-RAC2 and RAC2-to-RAC1 clearly affected their interaction with GDI1 (Figure 3B,C). This notion goes along with our other data and confirms the central role of HVR in the interaction with GDIs.

Spatiotemporal activation of RHO proteins requires that various regulators are orchestrated. GDI as a membrane cycling factor regulates the state of activation of RHO proteins by displacing them from different membranes and masking them from their activation by GEFs or proteasomal degradation [13,59]. Specific binding of the GDI proteins toward RAC1 in the terms of fast-kinetic elucidates a new mechanism through electrostatic steering between HVR of RAC1 and negatively charged N-terminus of GDI (Figure 5). Nevertheless, HVR determines the kinetics of membrane localization of RHO GTPases [48]. There are genuine data, which showed RAC1 with a strong polybasic region that would be mainly targeted to plasma membrane, but RAC2 and CDC42 with a weaker polybasic region remain in endomembranes [60]. In this context, the high affinity of RAC1 membrane binding calls for a tighter interaction with GDI in order to extract it from membranes.

A number of modulatory proteins and enzyme activities may facilitate or block the GDI-regulated RAC1 extraction from the membrane. A group of proteins that associate with the C-terminal HVR of RAC1 and may interfere with the RhoGDI function, includes CMS/CD2AP [46], β-PIX [61], Pacsin2 [62], NMP1 [63], smgGDS [64], and CaM [65]. The dissociation of RAC1 from GDI1 and its (re)association with the cell membrane still remain unclear.

There are several modulators proposed to fulfill these functions. Potential RhoGDI displacement factors include the neurotrophin receptor p75 (p75^NTR^) and TROY [66,67,68], and members of the ezrin/radixin/moesin (ERM) protein family [69,70,71]. Other factors that directly modulate the RhoGDI functions are coronin-1A [72,73], syndecan 4-synectin complex [74], the GAP domain of the RHO regulators BCR and ABR [75,76] and phospholipids, such as phosphoinositide (3,4,5)-trisphosphate (PIP_3_) [77]. Phosphorylation of GDI1 by PAK1 at Ser101 and Ser174 has been shown to mediate its dissociation from RAC1 but not RHOA [78]. However, the mechanistic details of such GDI modulators or displacement factors remain unclear.

Enzyme activities that control posttranslational modifications, including phosphorylation, acetylation and sumoylation, add an additional level of complexity to cellular biochemistry and the regulation of the GDI function. 14-3-3τ, a member of the 14-3-3 family, has been shown to promote tumor cell invasion and metastasis by binding to and inhibiting GDI1 [23]. 14-3-3τ binds phosphorylated GDI1 and interferes with its association with RHO proteins, thereby promoting epidermal growth factor (EGF)-induced RHO protein activation. GDI acetylation has also been shown to affect the RHO GTPase-GDI interactions [79], while GDI sumoylation increases this interaction [79,80], which is negatively regulated by the physical interaction of XIAP with GDI [81]. Ubiquitination of GDI by GRAIL, an E3 ligase, does not lead to proteolytic degradation but rather to stabilization of GDI [82].

The structure of uncomplexed GDI1 is yet unknown, raising yet unresolved questions about how the flexible NTA of GDI1 is stabilized and whether GDI1 undergoes conformational changes to open up the hydrophobic cavity of its GGBD.

Very recently, we identified a novel motif in the very C-terminal end of GRB2, consisting of four amino acids, that appears to play a key role in the allosteric regulation of GRB2 signaling from activated receptors to SOS1 activation [83]. It is remarkable that deletion of the CT motif of GDI1 completely abolished the GDI1-induced displacement of RAC1 from the liposomes. We propose that this motif physically binds terminal amino acids of GDI1 NTA and ultimately locks the formed RAC1^GG^-GDI1 complex. However, the CT motif may also contribute to the electrostatic pincer function, by pulling RAC1^GG^ out of the membrane.

While the RAC1 G domain mediates regulation and signaling [4], its HVR, modulated by various posttranslational modifications, finetunes intracellular trafficking, compartmentalization, subcellular localization, interactions, and membrane association by interacting with a variety of proteins [46]. Given this knowledge and the results presented in this study, a crucial and interesting area for future research, is to examine modulatory mechanisms, controlling RAC1 function, which impinges on its C-terminal region. Last but not least, unraveling of the molecular basis of RAC1 regulation may aid in understanding a variety of diseases with the implication of RAC1 deregulation and dysfunction, including atherosclerosis, diabetes and cancer [84].

## 4. Materials and Methods

### 4.1. Constructs

Different variants pGEX vectors (pGEX2T and pGEX4T-1) encoding an N-terminal glutathione S-transferase (GST) fusion protein were used to overexpress human GDI1 (acc. no. D13989), GDI2 (acc. no. P52566), and GDI3 (acc. no. Q99819) as well as human RHO-related genes, i.e., RAC1 (acc. no. P63000; aa 1-179), RAC2 (acc. no. P15153; aa 1-192), RAC3 (acc. no. P60763; aa 1-192), RHOG (acc. no. P84095; aa 1-178), RHOA (acc. no. P61586; aa 1-181), RHOB (acc. no. P62745; aa 1-181), RHOC (acc. no. P08134; aa 1-181), CDC42 (acc. no. P60953; aa 1-178), TC10 (acc. no. P17081; aa 2-193), RIF (acc. no. Q9HBH0; aa 1-195), and mouse RHOD (acc. no. P97348; aa 2-193). For baculovirus-insect cell expression, human RAC1 was subcloned into pFastBacHTB vector (Invitrogen, Carlsbad, CA, USA) and fused with an N-terminal hexa-histidine (6xHis) tag. For expression in human cells, RAC1 and GDI1 variants were cloned in pEYFP and pcDNA-FLAG vectors. All RHO GTPase and GDI variants were generated by PCR-based site-directed mutagenesis as described [85].

### 4.2. Proteins

All proteins were produced using *Escherichia coli* and baculovirus-insect cell expression system as described [34]. Glutathione S-transferase (GST) fusion proteins were isolated by affinity chromatography on a glutathione Sepharose column in the first step and purified by size exclusion chromatography after proteolytic cleavage of GST in the second step [86]. His-tagged proteins were isolated from SF9 insect cells, using affinity chromatography on Ni-NTA columns. The quality of the proteins was analyzed by 12% SDS-PAGE. Protein concentrations were determined using Bradford reagent (Coomassie dye reagent; Sigma, (Steinheim, Germany)), and the GDP concentration in the case of purified RHO GTPases was determined using HPLC [87]. Nucleotide-free RHO proteins were prepared using alkaline phosphatase (Sigma Aldrich, Deisenhofen, Germany)and phosphodiesterase (Sigma Aldrich, Deisenhofen, Germany) at 4 °C as previously described [87]. RHO GTPases were loaded with 2-deoxy-3-O-N-methylanthraniloyl GDP (mdGDP); the fluorescent reporter group methyl-anthraniloyl (m) was attached to the 3′-OH group, and can, due to the lack 2′-OH, not isomerize between 2′- and 3-OH groups as compared to mGDP [88].

### 4.3. Liposome Assays

The liposomes were freshly prepared to perform liposome sedimentation as described [34]. Briefly, liposome assays were performed by mixing and incubating the liposomes and purified RAC1 proteins. The mixtures were incubated for different time points and centrifuged at different speeds to separate the liposome pellets and supernatants for optimizing the centrifuging force. The liposomes were prepared as a lipid mixture (194 μg), containing 39% (*w*/*w*) phosphatidylethanolamine (PE), 16% (*w*/*w*) phosphatidylcholine (PC), 36% (*w*/*w*) phosphatidylserine (PS), 4% (*w*/*w*) sphingomyelin (SM), and 5% (*w*/*w*) phosphatidylinositol 4,5-bisphosphate (PIP_2_), and phosphatidylinositol 3,4,5-trisphosphate (PIP_3_), that was dried using light nitrogen stream. The lipids were purchased from Sigma-Aldrich (Munich, Germany). Obtained lipid film was hydrated with 300 μL of a buffer, containing 30 mM HEPES-NaOH pH 7.4, 50 mM NaCl, 3 mM DTT, 5 mM MgCl_2_. Sonication (20 s with minimal power, 50% off and 50% on) was employed finally to form liposomes, which were ultimately extruded through a filter with a pore size of 0.2 µm.

### 4.4. Fluorescence Measurements

Kinetics and equilibrium measurements were performed as described [86]. Briefly, all fluorescence measurements were performed at 25 °C in buffer containing 30 mM Tris/HCl, pH 7.5, 10 mM K_2_HPO_4_/KH_2_PO_4_, pH 7.5, 5 mM MgCl_2_, and 3 mM Dithiothreitol (DTT). The association of mdGDP-bound RHO GTPases (0.2 µM) with RHOGDIs (2 µM or increasing concentrations) was measured in a time-dependent manner using a stopped-flow instrument SF-61, HiTech Scientific (TgK Scientific Limited, Bradford, UK) and SX20 MV, Applied Photophysics (Leatherhead, UK). Emission was detected through a cutoff filter of 408 nm. The observed rate constants were calculated by fitting the data as single exponential decay using the GraFit program (Erithacus software, Staines, UK). Dissociation experiments were performed by displacing the bound GDI from the complex upon adding excess unlabeled GDP-bound RHO proteins. Fluorescence polarization experiments were performed in a Fluoromax 4 fluorimeter (Horiba Jobin Yvon, France) in polarization mode by titrating increasing amounts of different variants of GDI1 to mdGDP-bound RHO proteins (0.2 µM) in a total volume of 200 µL. An excitation wavelength of 360 nm and an emission wavelength of 450 nm were used. The Kd values were calculated by fitting the concentration-dependent binding curve using a quadratic ligand binding equation.

### 4.5. Sequence and Structural Analysis

Sequence alignments were performed with the BioEdit program using the ClustalW algorithm [89]. The intermolecular contacts were determined (<4.0 Å) between the GDIs and RHO GTPases using available RHO GTPase-GDI complex structures in the Protein Data Bank. A python code has been written using BioPython modules (pairwise2 and SubsMat.MatrixInfo) [90] to calculate inter-molecular distances in PDB structures between the pairs of residues as interaction matrix and synchronise them with sequence alignments of RHO GTPases and GDIs respectively. All structural representations were generated using PyMOL viewer [91].

### 4.6. Nucleofection and Immunofluorescence Analysis

pYFP-RAC1 and pcDNA3-FLAG-GDI1 plasmids were microporated into HUVECs using the 4D-NucleofectorTM system (Lonza, Cologne, Germany) according to the manufacturer’s instructions. After transfection, cells were seeded on fibronectin-coated (5 μg/mL) 2 cm^2^ glass coverslips (Thermo Scientific, Menzel-gläser, Germany) and were fixed after 24 h for immunofluorescence analysis. After fixing with warm (37 °C) 4% paraformaldehyde in phosphate buffered saline (PBS) for 15 min followed by three washes with PBS, coverslips were mounted on Mowiol4-88/DABCO solution (Calbiochem, Sigma Aldrich). Cells were permeabilized with 0,2% triton X-100 in PBS for 3 min and blocked for 30 min with 1% HSA in PBS. Hereafter, coverslips were stained with primary anti-FLAG antibody (Sigma, # F7425) in 1% HSA/PBS over night at 4 °C. After washing with PBS, coverslips were incubated with Alexa^®^ 555-conjugated anti-rabbit antibody (# A-31572; Scientific Life Technologies, Darmstadt, Germany) for 1 h at room temperature. Coverslips were mounted with Mowiol4-88/DABCO solution (Calbiochem, Sigma Aldrich). Confocal scanning laser microscopy was performed on a Nikon A1R confocal microscope (Nikon Instruments, Amsterdam, The Netherlands). Cells with moderate expression of the constructs and no aberrant phenotype from non-transfected neighboring cells were imaged. A z-stack image with a total thickness of 2 µm was acquired and images were equally adjusted using ImageJ (Li-CORE Biosciences (Bad Homburg, Germany). The max z-projection is shown with a scale bar representing 50 µm.

## Data Availability

This study includes no data deposited in external repositories.

## Supplementary Materials

The following are available online at https://www.mdpi.com/article/10.3390/ijms222212493/s1.

## References

[1] Wennerberg K., Der C.J. (2004). Rho-family GTPases: It’s not only Rac and Rho (and I like it). J. Cell Sci..

[2] Roberts P.J., Mitin N., Keller P.J., Chenette E.J., Madigan J.P., Currin R.O., Cox A.D., Wilson O., Kirschmeier P., Der C.J. (2008). Rho family GTPase modification and dependence on CAAX motif-signaled posttranslational modification. J. Biol. Chem..

[3] Ahmadian M.R., Jaiswal M., Fansa E.K., Dvorsky R. (2013). New insight into the molecular switch mechanism of human Rho family proteins: Shifting a paradigm. Biol. Chem..

[4] Dvorsky R., Ahmadian M.R. (2004). Always look on the bright site of Rho: Structural implications for a conserved intermolecular interface. EMBO Rep..

[5] Cherfils J., Zeghouf M. (2013). Regulation of small GTPases by GEFs, GAPs, and GDIs. Physiol. Rev..

[6] Bishop A.L., Hall A. (2000). Rho GTPases and their effector proteins. Biochem. J..

[7] White C.D., Erdemir H.H., Sacks D.B. (2012). IQGAP1 and its binding proteins control diverse biological functions. Cell. Signal..

[8] Watanabe T., Wang S., Kaibuchi K. (2015). IQGAPs as Key Regulators of Actin-cytoskeleton Dynamics Mini-review and Review. Cell Struct. Funct..

[9] Abel A.M., Schuldt K.M., Rajasekaran K., Hwang D., Riese M.J., Rao S., Thakar M.S., Malarkannan S. (2015). IQGAP1: Insights into the function of a molecular puppeteer. Mol. Immunol..

[10] Heasman S.J., Ridley A.J. (2008). Mammalian Rho GTPases: New insights into their functions from in vivo studies. Nat. Rev. Mol. Cell Biol..

[11] Hedman A.C., Smith J.M., Sacks D.B. (2015). The biology of IQGAP proteins: Beyond the cytoskeleton. EMBO Rep..

[12] Hall A. (2012). Rho family GTPases. Biochemical Society Transactions.

[13] Garcia-Mata R., Boulter E., Burridge K. (2011). The “invisible hand”: Regulation of RHO GTPases by RHOGDIs. Nat. Rev. Mol. Cell Biol..

[14] Jaiswal M., Dvorsky R., Ahmadian M.R. (2013). Deciphering the molecular and functional basis of Dbl family proteins: A novel systematic approach toward classification of selective activation of the Rho family proteins. J. Biol. Chem..

[15] Amin E., Jaiswal M., Derewenda U., Reis K., Nouri K., Koessmeier K.T., Aspenström P., Somlyo A.V., Dvorsky R., Ahmadian M.R. (2016). Deciphering the molecular and functional basis of RHOGAP family proteins: A systematic approach toward selective inactivation of RHO family proteins. J. Biol. Chem..

[16] Dovas A., Couchman J.R. (2005). RhoGDI: Multiple functions in the regulation of Rho family GTPase activities. Biochem. J..

[17] Xie F., Shao S., Aziz A.U.R., Zhang B., Wang H., Liu B. (2017). Role of Rho-specific guanine nucleotide dissociation inhibitor α regulation in cell migration. Acta Histochem..

[18] Griner E.M., Theodorescu D. (2012). The faces and friends of RhoGDI2. Cancer Metastasis Rev..

[19] De León-Bautista M.P., del Carmen Cardenas-Aguayo M., Casique-Aguirre D., Almaraz-Salinas M., Parraguirre-Martinez S., Olivo-Diaz A., del Rocío Thompson-Bonilla M., Vargas M. (2016). Immunological and functional characterization of RhoGDI3 and its molecular targets RhoG and RhoB in human pancreatic cancerous and normal cells. PLoS ONE.

[20] Brunet N., Morin A., Olofsson B. (2002). RhoGDI-3 regulates RhoG and targets this protein to the Golgi complex through its unique N-terminal domain. Traffic.

[21] Ahmad Mokhtar A.M.B., Ahmed S.B.M., Darling N.J., Harris M., Mott H.R., Owen D. (2021). A Complete Survey of RhoGDI Targets Reveals Novel Interactions with Atypical Small GTPases. Biochemistry.

[22] Ueyama T., Son J., Kobayashi T., Hamada T., Nakamura T., Sakaguchi H., Shirafuji T., Saito N. (2013). Negative Charges in the Flexible N-Terminal Domain of Rho GDP-Dissociation Inhibitors (RhoGDIs) Regulate the Targeting of the RhoGDI–Rac1 Complex to Membranes. J. Immunol..

[23] Xiao Y., Lin V.Y., Ke S., Lin G.E., Lin F.-T., Lin W.-C. (2014). 14-3-3 Promotes Breast Cancer Invasion and Metastasis by Inhibiting RhoGDI. Mol. Cell. Biol..

[24] Harding M.A., Theodorescu D. (2010). RhoGDI signaling provides targets for cancer therapy. Eur. J. Cancer.

[25] Moissoglu K., Schwartz M.A. (2014). Spatial and temporal control of Rho GTPase functions. Cell. Logist..

[26] Hodge R.G., Ridley A.J. (2016). Regulating Rho GTPases and their regulators. Nat. Rev. Mol. Cell Biol..

[27] DerMardirossian C., Bokoch G.M. (2005). GDIs: Central regulatory molecules in Rho GTPase activation. Trends Cell Biol..

[28] Dharmaiah S., Bindu L., Tran T.H., Gillette W.K., Frank P.H., Ghirlando R., Nissley D.V., Esposito D., McCormick F., Stephen A.G. (2016). Structural basis of recognition of farnesylated and methylated KRAS4b by PDEd. Proc. Natl. Acad. Sci. USA.

[29] Weise K., Kapoor S., Werkmüller A., Möbitz S., Zimmermann G., Triola G., Waldmann H., Winter R. (2012). Dissociation of the K-Ras4B/PDEδ complex upon contact with lipid membranes: Membrane delivery instead of extraction. J. Am. Chem. Soc..

[30] Ismail S.A., Chen Y.X., Rusinova A., Chandra A., Bierbaum M., Gremer L., Triola G., Waldmann H., Bastiaens P.I.H., Wittinghofer A. (2011). Arl2-GTP and Arl3-GTP regulate a GDI-like transport system for farnesylated cargo. Nat. Chem. Biol..

[31] Chandra A., Grecco H.E., Pisupati V., Perera D., Cassidy L., Skoulidis F., Ismail S.A., Hedberg C., Hanzal-Bayer M., Venkitaraman A.R. (2012). The GDI-like solubilizing factor PDEδ sustains the spatial organization and signalling of Ras family proteins. Nat. Cell Biol..

[32] Tnimov Z., Abankwa D., Alexandrov K. (2014). RhoGDI facilitates geranylgeranyltransferase-I-mediated RhoA prenylation. Biochem. Biophys. Res. Commun..

[33] Robbe K., Otto-Bruc A., Chardin P., Antonny B. (2003). Dissociation of GDP dissociation inhibitor and membrane translocation are required for efficient activation of Rac by the Dbl homology-pleckstrin homology region of Tiam. J. Biol. Chem..

[34] Zhang S.C., Gremer L., Heise H., Janning P., Shymanets A., Cirstea I.C., Krause E., Nürnberg B., Ahmadian M.R. (2014). Liposome reconstitution and modulation of recombinant prenylated human Rac1 by GEFs, GDI1 and Pak1. PLoS ONE.

[35] Grizot S., Fauré J., Fieschi F., Vignais P.V., Dagher M.C., Pebay-Peyroula E. (2001). Crystal structure of the Rac1—RhoGDI complex involved in NADPH oxidase activation. Biochemistry.

[36] Scheffzek K., Stephan I., Jensen O.N., Illenberger D., Gierschik P. (2000). The Rac-RhoGDI complex and the structural basis for the regulation of Rho proteins by RhoGDI. Nat. Struct. Biol..

[37] Dransart E., Olofsson B., Cherfils J. (2005). RhoGDIs revisited: Novel roles in Rho regulation. Traffic.

[38] Hoffman G.R., Nassar N., Cerione R.A. (2000). Structure of the Rho family GTP-binding protein Cdc42 in complex with the multifunctional regulator RhoGDI. Cell.

[39] Longenecker K., Read P., Derewenda U., Dauter Z., Liu X., Garrard S., Walker L., Somlyo A.V., Nakamoto R.K., Somlyo A.P. (1999). How RhoGDI binds Rho. Acta Crystallogr. Sect. D Biol. Crystallogr..

[40] Johnson J.L., Erickson J.W., Cerione R.A. (2009). New insights into how the Rho guanine nucleotide dissociation inhibitor regulates the interaction of Cdc42 with membranes. J. Biol. Chem..

[41] Newcombe A.R., Stockley R.W., Hunter J.L., Webb M.R. (1999). The Interaction between Rac1 and Its Guanine Nucleotide Dissociation Inhibitor (GDI), Monitored by a Single Fluorescent Coumarin Attached to GDI. Biochemistry.

[42] Tnimov Z., Guo Z., Gambin Y., Nguyen U.T.T., Wu Y.W., Abankwa D., Stigter A., Collins B.M., Waldmann H., Goody R.S. (2012). Quantitative analysis of prenylated RhoA interaction with its chaperone, RhoGDI. J. Biol. Chem..

[43] Haeusler L.C., Hemsath L., Fiegen D., Blumenstein L., Herbrand U., Stege P., Dvorsky R., Ahmadian M.R. (2006). Purification and biochemical properties of Rac1, 2, 3 and the splice variant Rac1b. Methods Enzymol..

[44] Haeusler L.C., Blumenstein L., Stege P., Dvorsky R., Ahmadian M.R. (2003). Comparative functional analysis of the Rac GTPases. FEBS Lett..

[45] Golovanov A.P., Hawkins D., Barsukov I., Badii R., Bokoch G.M., Lian L.-Y., Roberts G.C.K. (2001). Structural consequences of site-directed mutagenesis in flexible protein domains. Eur. J. Biochem..

[46] Lam B.D., Hordijk P.L. (2013). The Rac1 hyper variable region in targeting and signaling-a tail of many stories. Small GTPases.

[47] Joseph G., Gorzalczany Y., Koshkin V., Pick E. (1994). Inhibition of NADPH oxidase activation by synthetic peptides mapping within the carboxyl-terminal domain of small GTP-binding proteins. Lack of amino acid sequence specificity and importance of polybasic motif. J. Biol. Chem..

[48] Maxwell K.N., Zhou Y., Hancock J.F. (2018). Rac1 Nanoscale Organization on the Plasma Membrane Is Driven by Lipid Binding Specificity Encoded in the Membrane Anchor. Mol. Cell. Biol..

[49] Gosser Y.Q., Nomanbhoy T.K., Aghazadeh B., Manor D., Combs C., Cerione R.A., Rosen M.K. (1997). C-terminal binding domain of Rho GDP-dissociation inhibitor directs N-terminal inhibitory peptide to GTPases. Nature.

[50] Forget M.A., Desrosiers R.R., Gingras D., Béliveau R. (2002). Phosphorylation states of Cdc42 and RhoA regulate their interactions with Rho GDP dissociation inhibitor and their extraction from biological membranes. Biochem. J..

[51] Golovanov A.P., Chuang T.H., DerMardirossian C., Barsukov I., Hawkins D., Badii R., Bokoch G.M., Lian L.Y., Roberts G.C.K. (2001). Structure-activity relationships in flexible protein domains: Regulation of rho GTPases by RhoGDI and D4 GDI. J. Mol. Biol..

[52] Lam M.T., Coppola S., Krumbach O.H.F., Prencipe G., Insalaco A., Cifaldi C., Brigida I., Zara E., Scala S., di Cesare S. (2019). A novel disorder involving dyshematopoiesis, inflammation, and HLH due to aberrant CDC42 function. J. Exp. Med..

[53] Keep N.H., Barnes M., Barsukov I., Badii R., Lian L.Y., Segal A.W., Moody P.C.E., Roberts G.C.K. (1997). A modulator of rho family G proteins, rhoGDI, binds these G proteins via an immunoglobulin-like domain and a flexible N-terminal arm. Structure.

[54] Hemsath L., Dvorsky R., Fiegen D., Carlier M.F., Ahmadian M.R. (2005). An electrostatic steering mechanism of Cdc42 recognition by Wiskott-Aldrich syndrome proteins. Mol. Cell.

[55] Tetley G.J.N., Szeto A., Fountain A.J., Mott H.R., Owen D. (2018). Bond swapping from a charge cloud allows flexible coordination of upstream signals through WASP: Multiple regulatory roles for the WASP basic region. J. Biol. Chem..

[56] Jiang Y., Fu H., Springer T.A., Wong W.P. (2019). Electrostatic Steering Enables Flow-Activated Von Willebrand Factor to Bind Platelet Glycoprotein, Revealed by Single-Molecule Stretching and Imaging. J. Mol. Biol..

[57] Didsbury J., Weber R.F., Bokoch G.M., Evans T., Snyderman R. (1989). Rac, a Novel Ras-Related Family of Proteins That Are Botulinum Toxin Substrates. J. Biol. Chem..

[58] Scherle P., Behrens T., Staudt L.M. (1993). Ly-GDI, a GDP-dissociation inhibitor of the RhoA GTP-binding protein, is expressed preferentially in lymphocytes. Proc. Natl. Acad. Sci. USA.

[59] Majolée J., Podieh F., Hordijk P.L., Kovačević I. (2021). The interplay of Rac1 activity, ubiquitination and GDI binding and its consequences for endothelial cell spreading. PLoS ONE.

[60] Michaelson D., Silletti J., Murphy G., D’Eustachio P., Rush M., Philips M.R. (2001). Differential localization of Rho GTPases in live cells: Regulation by hypervariable regions and RhoGDI binding. J. Cell Biol..

[61] Ten Klooster J.P., Jaffer Z.M., Chernoff J., Hordijk P.L. (2006). Targeting and activation of Rac1 are mediated by the exchange factor β-Pix. J. Cell Biol..

[62] De Kreuk B.J., Nethe M., Fernandez-Borja M., Anthony E.C., Hensbergen P.J., Deelder A.M., Plomann M., Hordijk P.L. (2011). The F-BAR domain protein PACSIN2 associates with Rac1 and regulates cell spreading and migration. J. Cell Sci..

[63] Zoughlami Y., van Stalborgh A.M., van Hennik P.B., Hordijk P.L. (2013). Nucleophosmin1 Is a Negative Regulator of the Small GTPase Rac1. PLoS ONE.

[64] Lanning C.C., Ruiz-Velasco R., Williams C.L. (2003). Novel mechanism of the co-regulation of nuclear transport of SmgGDS and Rac1. J. Biol. Chem..

[65] Xu B., Chelikani P., Bhullar R.P. (2012). Characterization and Functional Analysis of the Calmodulin-Binding Domain of Rac1 GTPase. PLoS ONE.

[66] Yamashita T., Tohyama M. (2003). The p75 receptor acts as a displacement factor that releases Rho from Rho-GDI. Nat. Neurosci..

[67] Lin Z., Tann J.Y., Goh E.T., Kelly C., Lim K.B., Gao J.F., Ibanez C.F. (2015). Structural basis of death domain signaling in the p75 neurotrophin receptor. Elife.

[68] Lu Y., Liu X., Zhou J., Huang A., Zhou J., He C. (2013). TROY interacts with Rho guanine nucleotide dissociation inhibitor α (RhoGDIα) to mediate Nogo-induced inhibition of neurite outgrowth. J. Biol. Chem..

[69] Scoles D.R. (2008). The merlin interacting proteins reveal multiple targets for NF2 therapy. Biochim. Biophys. Acta.

[70] Takahashi K., Sasaki T., Mammoto A., Takaishi K., Kameyama T., Tsukita S., Tsukita S., Takai Y. (1997). Direct interaction of the Rho GDP dissociation inhibitor with ezrin/radixin/moesin initiates the activation of the Rho small G protein. J. Biol. Chem..

[71] Maeda M., Matsui T., Imamura M., Tsukita S., Tsukita S. (1999). Expression level, subcellular distribution and Rho-GDI binding affinity of merlin in comparison with ezrin/radixin/moesin proteins. Oncogene.

[72] Castro-Castro A., Ojeda V., Barreira M., Sauzeau V., Navarro-Lérida I., Muriel O., Couceiro J.R., Pimentel-Muíños F.X., del Pozo M.A., Bustelo X.R. (2011). Coronin 1A promotes a cytoskeletal-based feedback loop that facilitates Rac1 translocation and activation. EMBO J..

[73] Castro-Castro A., Muriel O., del Pozo M.A., Bustelo X.R. (2016). Characterization of Novel Molecular Mechanisms Favoring Rac1 Membrane Translocation. PLoS ONE.

[74] Elfenbein A., Rhodes J.M., Meller J., Schwartz M.A., Matsuda M., Simons M. (2009). Suppression of RhoG activity is mediated by a syndecan 4-synectin-RhoGDI1 complex and is reversed by PKCα in a Rac1 activation pathway. J. Cell Biol..

[75] Kweon S.-M., Cho Y.J., Minoo P., Groffen J., Heisterkamp N. (2008). Activity of the Bcr GTPase-activating domain is regulated through direct protein/protein interaction with the Rho guanine nucleotide dissociation inhibitor. J. Biol. Chem..

[76] Ota T., Maeda M., Okamoto M., Tatsuka M. (2015). Positive regulation of Rho GTPase activity by RhoGDIs as a result of their direct interaction with GAPs. BMC Syst. Biol..

[77] Ugolev Y., Berdichevsky Y., Weinbaum C., Pick E. (2008). Dissociation of Rac1(GDP).RhoGDI complexes by the cooperative action of anionic liposomes containing phosphatidylinositol 3,4,5-trisphosphate, Rac guanine nucleotide exchange factor, and GTP. J. Biol. Chem..

[78] Der Mardirossian C., Schnelzer A., Bokoch G.M. (2004). Phosphorylation of RhoGDI by Pak1 mediates dissociation of Rac GTPase. Mol. Cell.

[79] Kuhlmann N., Wroblowski S., Knyphausen P., de Boor S., Brenig J., Zienert A.Y., Meyer-Teschendorf K., Praefcke G.J.K., Nolte H., Krüger M. (2016). Structural and Mechanistic Insights into the Regulation of the Fundamental Rho Regulator RhoGDIα by Lysine Acetylation. J. Biol. Chem..

[80] Yu J., Zhang D., Liu J., Li J., Yu Y., Wu X.-R., Huang C. (2012). RhoGDI SUMOylation at Lys-138 increases its binding activity to Rho GTPase and its inhibiting cancer cell motility. J. Biol. Chem..

[81] Liu J., Zhang D., Luo W., Yu Y., Yu J., Li J., Zhang X., Zhang B., Chen J., Wu X.-R. (2011). X-linked inhibitor of apoptosis protein (XIAP) mediates cancer cell motility via Rho GDP dissociation inhibitor (RhoGDI)-dependent regulation of the cytoskeleton. J. Biol. Chem..

[82] Su L., Lineberry N., Huh Y., Soares L., Fathman C.G. (2006). A novel E3 ubiquitin ligase substrate screen identifies Rho guanine dissociation inhibitor as a substrate of gene related to anergy in lymphocytes. J. Immunol..

[83] Jasemi N.S.K., Herrmann C., Estirado E.M., Gremer L., Willbold D., Brunsveld L., Dvorsky R., Ahmadian M.R. (2021). The intramolecular allostery of GRB2 governing its interaction with SOS1 is modulated by phosphotyrosine ligands. Biochem. J..

[84] Olson M.F. (2018). Rho GTPases, their post-translational modifications, disease-associated mutations and pharmacological inhibitors. Small GTPases.

[85] Ahmadian M.R., Stege P., Scheffzek K., Wittinghofer A. (1997). Confirmation of the arginine-finger hypothesis for the GAP-stimulated GTP-hydrolysis reaction of Ras. Nat. Struct. Biol..

[86] Hemsath L., Ahmadian M.R. (2005). Fluorescence approaches for monitoring interactions of Rho GTPases with nucleotides, regulators, and effectors. Methods.

[87] Eberth A., Ahmadian M.R. (2009). In vitro GEF and GAP assays. Curr. Protoc. Cell Biol..

[88] John J., Sohmen R., Feuerstein J., Linke R., Wittinghofer A., Goody R.S. (2002). Kinetics of interaction of nucleotides with nucleotide-free H-ras p21. Biochemistry.

[89] Hall T.A. (1999). BioEdit A User-Friendly Biological Sequence Alignment Editor and Analysis Program for Windows 95/98/NT. Nucleic Acids Symposium Series, 41, 95–98. References—Scientific Research Publishing. https://www.scirp.org/(S(lz5mqp453edsnp55rrgjct55))/reference/ReferencesPapers.aspx?ReferenceID=1383440.

[90] Cock P.J.A., Antao T., Chang J.T., Chapman B.A., Cox C.J., Dalke A., Friedberg I., Hamelryck T., Kauff F., Wilczynski B. (2009). Biopython: Freely available Python tools for computational molecular biology and bioinformatics. Bioinformatics.

[91] DeLano W.L. (2002). The PyMOL Molecular Graphics System. Delano Scientific, San Carlos. References—Scientific Research Publishing. https://www.scirp.org/(S(vtj3fa45qm1ean45vvffcz55))/reference/ReferencesPapers.aspx?ReferenceID=1958992.

