# Supplementary material for: Electrostatic Forces Mediate the Specificity of RHO GTPase-GDI Interactions
Source: Int J Mol Sci. 2021 Nov 19;22(22):12493. doi: 10.3390/ijms222212493 (PMC8622166; doi:10.3390/ijms222212493)
Supplement: Supplementary file 1 [file ijms-22-12493-s001.zip › ijms-1456667-supplementary.pdf]

Supplementary Information

Electrostatic forces mediate the specificity of RHO GTPase-GDI interactions

Niloufar Mosaddeghzadeh, Neda S. Kazemein Jasemi, Jisca Majolée, Si-Cai Zhang, Peter L Hordijk, Radovan Dvorsky, M. Reza Ahmadian

Institute of Biochemistry and Molecular Biology II, Heinrich-Heine University, Düsseldorf, Germany.

Table S1. GDI1 and RHO GTPase variants used in this study.

Protein	Nomenclature	Variant description	Amino acids[a]	Uniprot ID
**GDI1**	$GDI1^{FL}$	Full-length	1-204	P52565
	$GDI1^{\Delta N15}$	N-terminal 15 aa truncation	16-204	
	$GDI1^{\Delta N25}$	N-terminal 25 aa truncation	26-204	
	$GDI1^{\Delta N58}$	N-terminal 58 aa truncation	59-198	
	$GDI1^{\Delta C6}$	C-terminal 6 aa truncation	1-198	
	$GDI1^{E121K}$	Glu121Lys	1-204	
	$GDI1^{FL(E163K/E164K)}$	Glu163Lys/ Glu164Lys	1-204	
	$GDI1^{FL(E140K/E163K/E164K)}$	Glu140Lys/ Glu163Lys/ Glu164Lys	1-204	
**RHO GTPase**	$RAC1^{GG}$	Geranylgeranylated RAC1	1-189	
	$RAC1^{FL}$	Full-length	1-192	P63000
	$RAC1^{FL(KRKRK\text{-}to\text{-}QQKRA)}$	RAC1 HVR to RAC2 HVR	1-192	
	$RAC1^{FL(KRKRK\text{-}to\text{-}EEEEE)}$	RAC1 HVR to negative residues	1-192	
	$RAC1^{\Delta C10}$	C-terminal 10 aa truncation	1-179	
	$RAC2^{FL}$	Full-length	1-192	P15153
	$RAC2^{FL(QQKRA\text{-}to\text{-}KRKRK)}$	RAC2 HVR to RAC1 HVR	1-192	
	$RAC3^{FL}$	Full-length	1-192	P60763
	$RHOG^{FL}$	Full-length	1-191	P84095
	$CDC42^{FL}$	Full-length	1-191	P60953
	$TC10^{FL}$	Full-length	1-213	P17081
	TCL^{FL}	Full-length	1-214	Q9H4E5
	$RHOA^{FL}$	Full-length	1-193	P61586
	$RHOB^{FL}$	Full-length	1-196	P62745
	$RHOC^{FL}$	Full-length	1-193	P08134
	$RHOD^{FL}$	Full-length	1-210	O00212
	RIF^{FL}	Full-length	1-211	Q9HBH0

Table S2. Published structures of the RHO GDIs alone and in the complex with RHO GTPase.

Structures[a]	PDB code	resolution (Å)	References[b]
GDI1	1GDF, 1AJW	NMR	(1)
GDI1	1RHO	2.50	(2)
$RHOA^{GG}$•GDP•GDI1	1CC0	5.00	(3)
$CDC42^{GG}$•GDP•GDI1	1DOA	2.60	(4)
$RAC2^{GG}$•GDP•GDI2	1DS6	2.35	(5)
$RHOA^{GG}$•GppNHp•GDI1	4F38	2.80	(6)
$RAC1^{GG}$•GDP•GDI1	1HH4	2.70	(7)
$RhoA^{Far}$•GDP•$GDI1^{Ac\text{-}K178}$	5FR2	3.35	(8)
$RHOA^{GG}$•GDP•$GDI1^{Ac\text{-}K127/Ac\text{-}K141}$	5FR1	2.75	(9)

[a] Ac, acetyl; Far, farnesyl; GG, geranylgeranyl.
[b] References are listed below.

References

1. Gosser, Y. Q., Nomanbhoy, T. K., Aghazadeh, B., Manor, D., Combs, C., Cerione, R. A., and Rosen, M. K. (1997) C-terminal binding domain of Rho GDP-dissociation inhibitor directs N-terminal inhibitory peptide to GTPases. *Nature* **387**, 814-819
2. Keep, N. H., Barnes, M., Barsukov, I., Badii, R., Lian, L.-Y., Segal, A. W., Moody, P. C. E., and Roberts, G. C. K. (1997) A modulator of rho family G proteins, rhoGDI, binds these G proteins via an immunoglobulin-like domain and a flexible N-terminal arm. *Structure* **5**, 623-633
3. Longenecker, K., Read, P., Derewenda, U., Dauter, Z., Liu, X., Garrard, S., Walker, L., Somlyo, A. V., Nakamoto, R. K., Somlyo, A. P., and Derewenda, Z. S. (1999) How RhoGDI binds Rho. *Acta Crystallogr D Biol Crystallogr* **55**, 1503-1515
4. Hoffman, G. R., Nassar, N., and Cerione, R. A. (2000) Structure of the Rho Family GTP-Binding Protein Cdc42 in Complex with the Multifunctional Regulator RhoGDI. *Cell* **100**, 345-356
5. Scheffzek, K., Stephan, I., Jensen, O. N., Illenberger, D., and Gierschik, P. (2000) The Rac-RhoGDI complex and the structural basis for the regulation of Rho proteins by RhoGDI. *Nat Struct Biol* **7**, 122-126
6. Tnimov, Z., Guo, Z., Gambin, Y., Nguyen, U. T., Wu, Y. W., Abankwa, D., Stigter, A., Collins, B. M., Waldmann, H., Goody, R. S., and Alexandrov, K. (2012) Quantitative analysis of prenylated RhoA interaction with its chaperone, RhoGDI. *J Biol Chem* **287**, 26549-26562
7. Grizot, S., Faure, J., Fieschi, F., Vignais, P. V., Dagher, M. C., and Pebay-Peyroula, E. (2001) Crystal structure of the Rac1-RhoGDI complex involved in nadph oxidase activation. *Biochemistry* **40**, 10007-10013
8. Kuhlmann, N., Wroblowski, S., Knyphausen, P., de Boor, S., Brenig, J., Zienert, A. Y., Meyer-Teschendorf, K., Praefcke, G. J., Nolte, H., Kruger, M., Schacherl, M., Baumann, U., James, L. C., Chin, J. W., and Lammers, M. (2016) Structural and Mechanistic Insights into the Regulation of the Fundamental Rho Regulator RhoGDIalpha by Lysine Acetylation. *J Biol Chem* **291**, 5484-5499
9. Kuhlmann, N., Wroblowski, S., Scislowski, L., and Lammers, M. (2016) RhoGDIalpha Acetylation at K127 and K141 Affects Binding toward Nonprenylated RhoA. *Biochemistry* **55**, 304-312

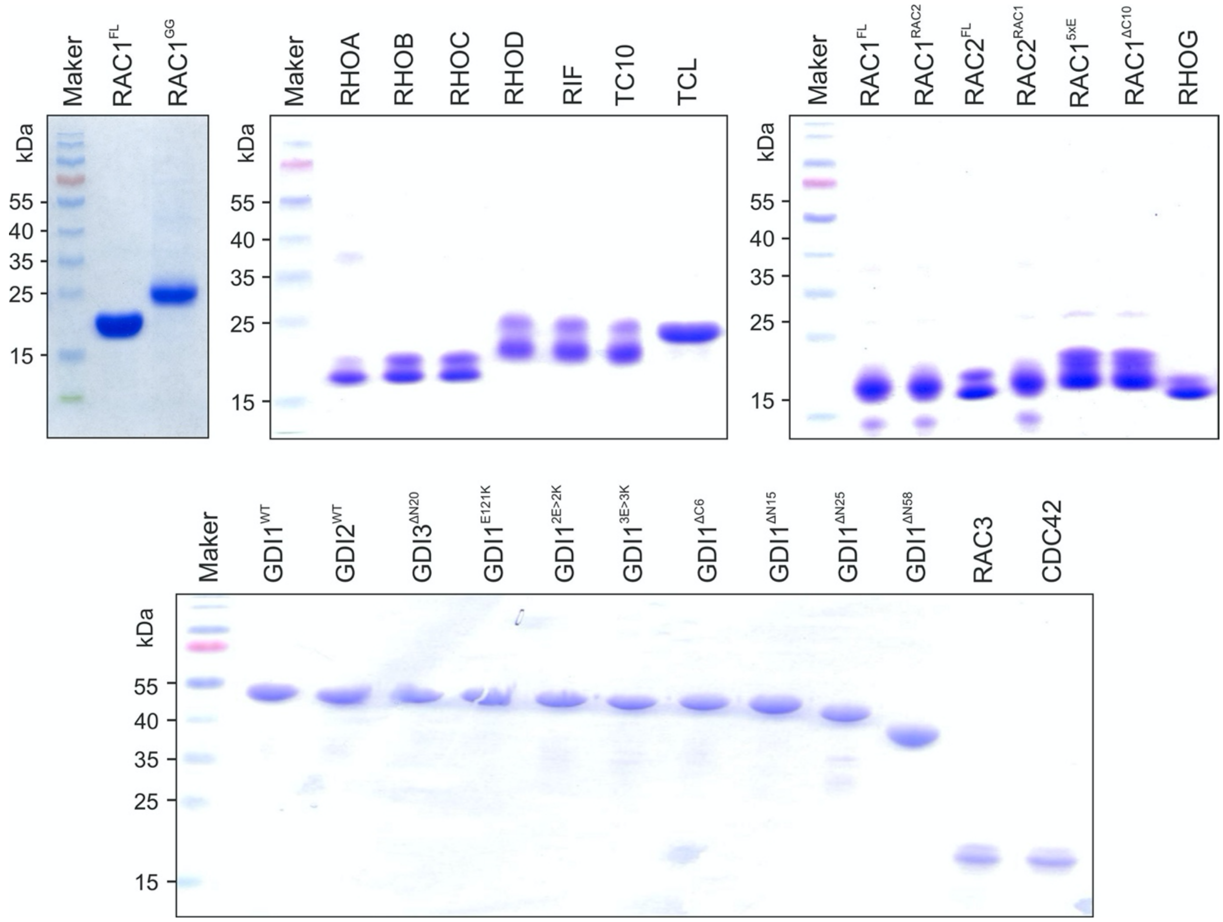

Figure S1. Sodium dodecyl sulfate polyacrylamide gel electrophoresis *(*SDS-PAGE*)* of proteins used in this study. SDS-PAGE was performed to show the purity of the analyzed RAC1 and GST fused-GDI proteins and their variants in this study. The gels are stained with Coomassie brilliant blue. The molecular mass from marker is indicated as kDa. kDa, kilodalton; FL, full-length; GG, geranylgeranyl moiety; M, molecular weight marker.

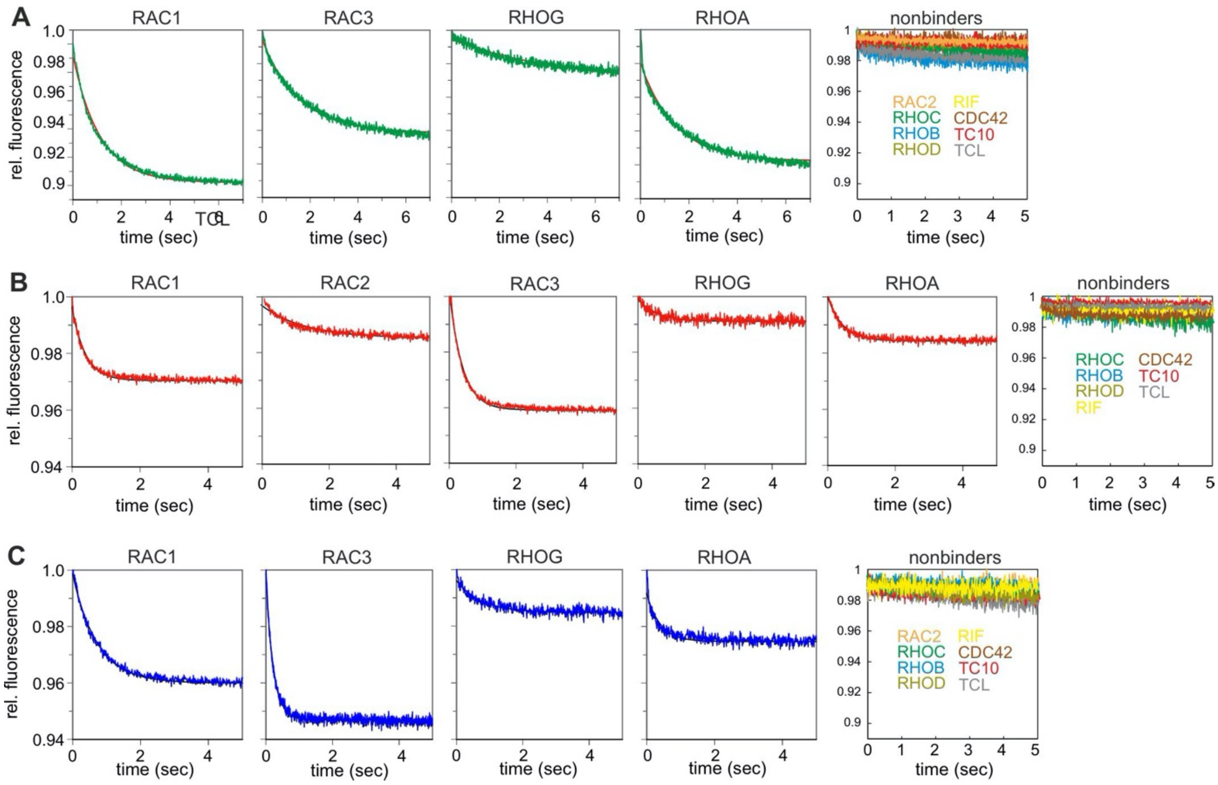

Figure S2. Kinetics of association of GDI1 (A), GDI2 (B) and GDI3 (C) with different members of the RHO GTPase family. GDIs (4 µM, respectively) was rapidly mixed with the mdGDP-bound RHO GTPases (0.2 µM, respectively) in a stopped-flow apparatus to monitor the association reaction between the two proteins. Observed rate constants (k_{obs}), obtained by fitting the data to single-exponential decay functions, are shown as bar charts in Figure 2A.

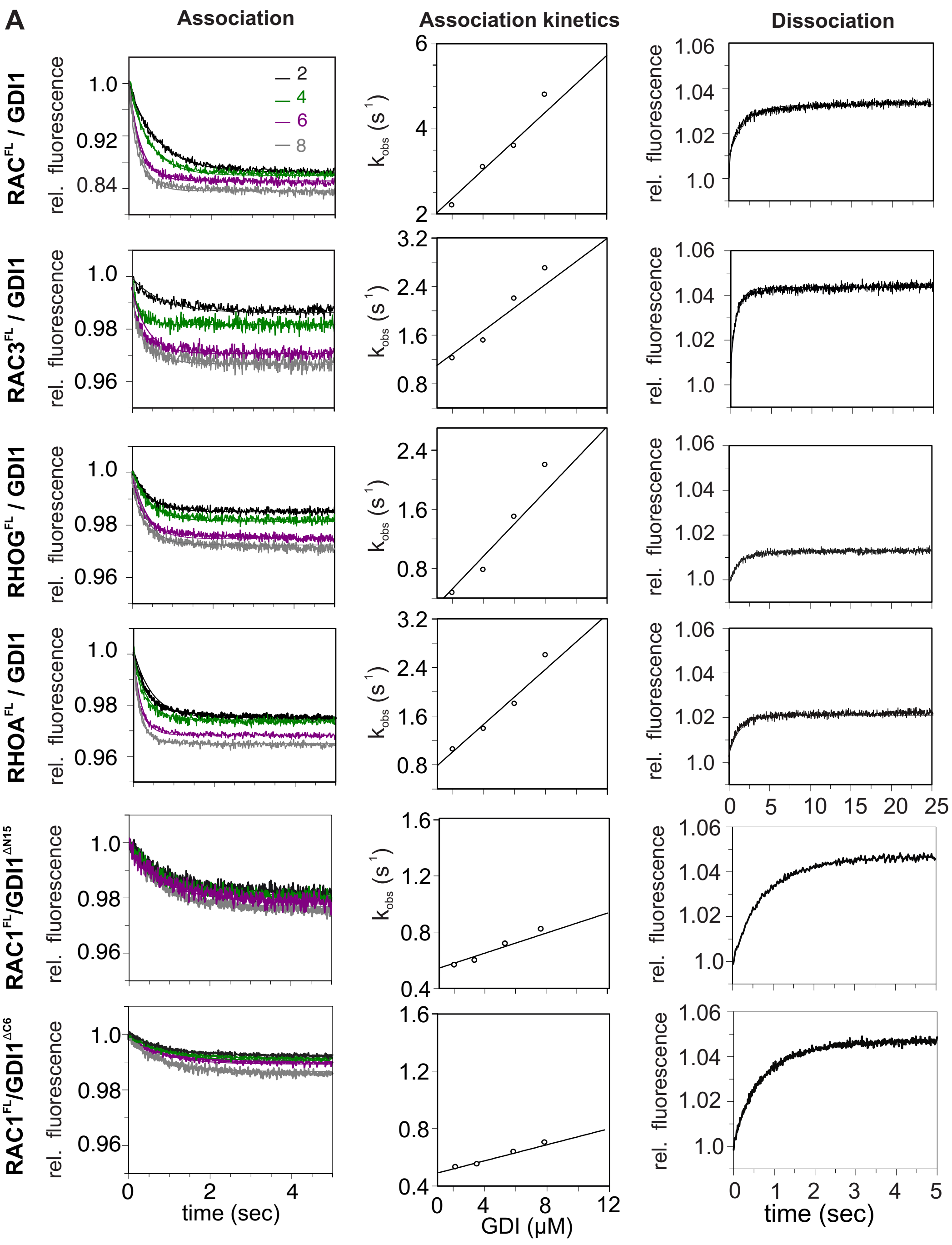

A
Association
Association kinetics
Dissociation
RAC^FL / GDI1
RAC3^FL / GDI1
RHOG^FL / GDI1
RHOA^FL / GDI1
RAC1^FL/GDI1^ΔN15
RAC1^FL/GDI1^ΔC6
rel. fluorescence
k_obs (s^-1)
2
4
6
8
time (sec)
GDI (μM)

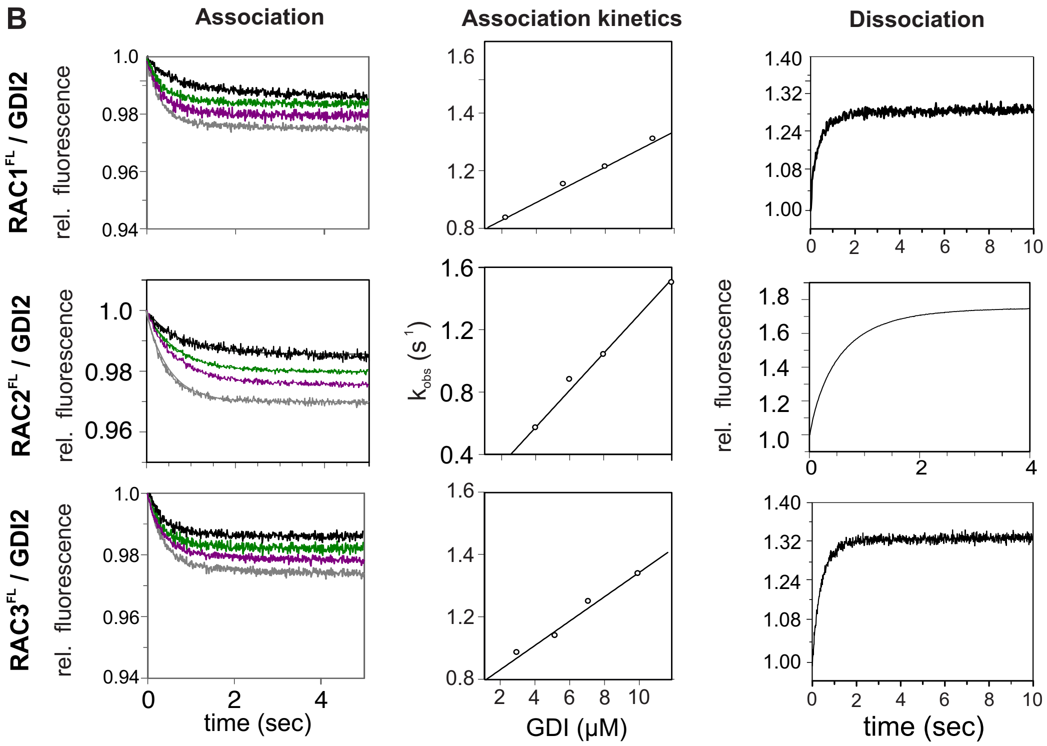

Figure S3. Kinetic measurements of the interactions with RHO GTPases with GDI1 (A) and GDI2 (B). Left panels: Association of mdGDP-bound GTPases (0.2 µM) with increasing GDI concentrations (2-8 µM). Middle panels: Evaluated association rate constant (k_{on}) from the plot of the k_{obs} values, obtained from the exponential fits to the association data (left panels) against the corresponding GDI concentrations. Right panels: Dissociation of GDI (2 µM) from mdGDP-bound GTPases (0.2 µM) in the presence of excess amounts of unlabeled GDP-bound GTPases (10 µM). The results are illustrated as bar charts in Figures 2B.

													17	23	25	26	27	28	29	30	31	32	33	34	38	41	42	45	46	47	48	49	50	51	52	53	54	55	56	110	121	122	124	125	127	130	140	141	142	143	144	145	146	147	148	149	150	158	160	161	162	163	164	166	167	184	185	IDs2	
GDI2 HS													D	S	L	N	Y	K	P	P	P	Q	K	S	L	M	D	D	E	S	L	I	K	Y	K	K	T	L	L	Y	D	I	S	G	K	Q	D	K	A	T	F	M	V	G	S	Y	G	F	T	P	V	E	E	P	K	D	D	P52566	
GDI1 HS													E	H	V	N	Y	K	P	P	A	Q	K	S	I	L	D	D	E	S	L	R	K	Y	K	E	A	L	L	Y	E	I	S	G	K	Q	D	K	T	D	Y	M	V	G	S	Y	G	F	T	P	V	E	E	P	K	D	D	P52565	
GDI3 HS															P	E	Y	R	A	P	G	R	K	S	I	L	D	D	R	S	L	A	K	Y	K	R	V	L	L	Y	E	I	S	G	K	H	D	K	T	V	Y	M	V	G	S	Y	G	F	T	P	V	E	E	P	R	D	D	Q99819	
GDI1 BT													E	H	V	N	Y	K	P	P	A	Q	K	S	I	L	D	D	E	S	L	R	K	Y	K	E	A	L	L	Y	E	I	S	G	K	Q	D	K	T	D	Y	M	V	G	S	Y	G	F	T	P	M	E	E	P	K	D	D	P19803	
GDI1 MM													E	H	V	N	Y	K	P	P	A	Q	K	S	I	L	D	D	E	S	L	R	K	Y	K	E	A	L	L	Y	E	I	S	G	K	Q	D	K	T	D	Y	M	V	G	S	Y	G	F	T	P	M	E	E	P	K	D	D	Q99PT1	
32	Y	Y	Y	Y	Y	Y	Y	Y	Y	Y	Y	Y																1	2	2																																								2	3
34	P	P	P	P	P	P	P	P	P	P	P	P																		5																																								5	1
35	T	T	T	T	T	T	T	T	T	T	T	T																6		7	4																																							7	3
36	V	V	V	V	V	V	V	V	V	V	V	V																1		7	5			6																																				7	4
37	F	F	F	F	F	F	F	F	F	F	F	F																		1				1																																				1	2
38	D	D	D	D	E	E	E	E	E	E	E	D																				2	4			2																																		4	3
39	N	N	N	N	N	N	N	N	N	N	N	N																					1				1																																	1	2
56	W	W	W	W	W	W	W	W	W	W	W	F																						1			1	1																																1	3
57	D	D	D	D	D	D	D	D	D	D	D	D																						6																																				6	1
58	T	T	T	T	T	T	T	T	T	T	T	T																						6																																				6	1
59	A	A	A	A	A	A	A	A	A	A	A	A																			1			7																																				7	2
60	G	G	G	G	G	G	G	G	G	G	G	G																						1																																				1	1
63	D	D	D	D	D	D	D	D	D	D	D	D										6		1	3																																											6	3
64	Y	Y	Y	Y	Y	Y	Y	Y	Y	Y	Y	Y										1		3	7	6				6		2	1	6																																		7	8
65	D	D	D	D	D	D	D	D	N	D	D	E							6						1	1				1				1																																		6	5
66	R	R	R	R	R	R	R	R	Q	R	R	R						1	7	8	8	5	2	1																	1	3	3																									1	6	8	12
67	L	L	L	L	L	L	L	L	L	L	L	L							1	1	1	1		3										3	5		1	6	4			3	1																										1	6	13
68	R	R	R	R	R	R	R	R	R	R	R	R												1										6	1			2	1																													6	5
69	P	P	P	P	P	P	P	P	P	P	P	T					1																	1																						8														8	3
70	L	L	L	L	L	L	L	L	L	L	L	L																										6	5			6														9	8	8												9	6
71	S	S	S	S	S	S	S	S	S	S	S	S																						1				5	1			1														1														5	5
72	Y	Y	Y	Y	Y	Y	Y	Y	Y	Y	Y	Y																										1																																1	1
73	P	P	P	P	P	P	P	P	P	P	P	P																																										1	1	4	6													6	4
74	Q	Q	Q	Q	D	D	D	D	D	D	D	Q																																												1	1													1	2
94	R	R	R	R	P	P	P	P	P	P	P	K				1																																																									1	1
95	A	A	A	A	E	E	E	E	E	E	E	E			1																																																									1	1
96	K	K	K	K	K	K	K	K	K	K	K	K			1			1																																																						1	2
98	Y	F	Y	Y	T	V	T	V	T	T	V	V		1	1																																																									1	2
99	P	P	P	P	P	P	P	P	P	P	P	P			3	3	5																																																							5	3
100	E	E	E	E	E	E	E	E	E	E	E	E			1		5																																																								5	2
101	V	V	V	V	V	V	V	V	V	V	V	I					1																																																							1	1
103	H	H	H	H	H	H	H	E	D	H	H	H			1		6																												3	2							8																8		8	6
104	H	H	H	H	F	F	F	Y	C	F	H	H					7																											1	5	1							6		2	8													5		8	8
105	C	C	C	C	C	C	C	Y	C	C	C	C					1																												1								1			1															1	4
106	P	P	P	P	P	P	P	P	P	P	P	P																																										4																	4	1
185	R	Q	P	R	K	K	R	C	G	T	K	P																																																				1	1	1					1	3
186	K	K	G	G	K	R	R	I	H	Q	R	K																																				1	1		1	1	1								1		3	1						3	8
187	R	R	K	R	G	Y	R	C	S	G	R	R	1																											1							1	2		2		2								1	1	1		3		1				3	11
188	K	A	K	S	S	G	G	N	C	F	L	K																																				1		1														1	1	2	1			2	6
	Rac1	Rac2	Rac3	RhoG	RhoA	RhoB	RhoC	TC10	TCL	RhoD	Rif	Cdc42	1	1	8	4	26	1	1	14	9	9	13	2	9	11	7	8	2	22	17	2	7	40	13	2	3	21	11	1	1	13	5	9	3	1	4	1	3	1	3	20	1	3	32	15	8	1	1	2	1	8	3	3	1	14	7		

Figure S4. Interaction matrix adapted for the structures of RHO GTPase complexes with the GDI proteins. Interaction matrix of RHO family proteins with the GDI proteins used in this study is generated to demonstrate interacting residues in respective structures (see Table S2). It comprises the amino acid sequence alignments of the RHO GTPases (lower left panel) and the GDI proteins (upper right panel), respectively. Each element of the matrix relates one homologous residue from RHO GTPases to one homologous residue from the GDI proteins. If at least one inter-molecular distance between the atoms of related residues is equal or smaller than 4Å these residues are considered as interacting. Each matrix element counts the number of such defined interactions in 9 analyzed complex structures between Rho GTPases and GDIs. Zero respectively empty cell means that corresponding residues do not contact each other in any structure while a maximal value 9 means that this particular interaction exists in all structures and is accounted as a \`hotspot´. RAC2 residues (Pro28, Leu35, Met38, Asp39, Ala139 and Phe141; GDI2 numbering) that are highlighted in red boxes, deviate from those in GDI1 and GDI3.

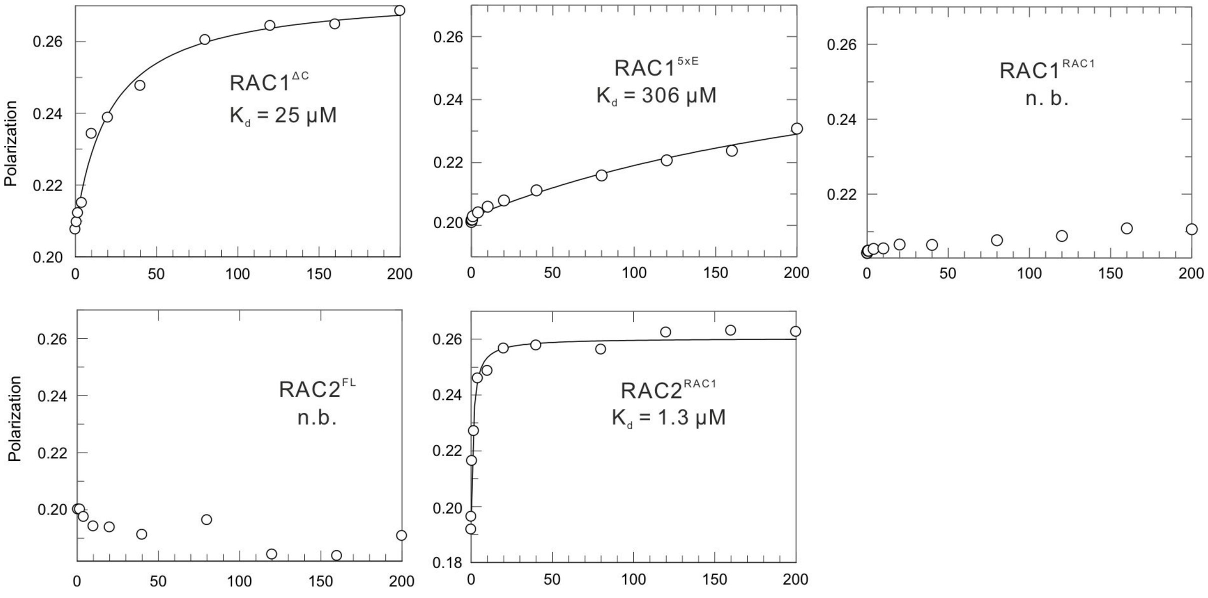

Figure S5. Fluorescence polarization measurements of GDI1^{WT} interactions with the RAC variants. Experiments were performed to determine the dissociation constants (K_d) for the interaction of increasing concentrations of GDI1 with various mdGDP-bound RAC variants as indicated. The X-axis represents the GDI1 concentration in µM and Y-axis represents relative fluorescence polarization. Equilibrium K_d values for the respective interactions were calculated by fitting data to a quadratic ligand-binding equation (solid lines). These data are summarized in Figure 3C.

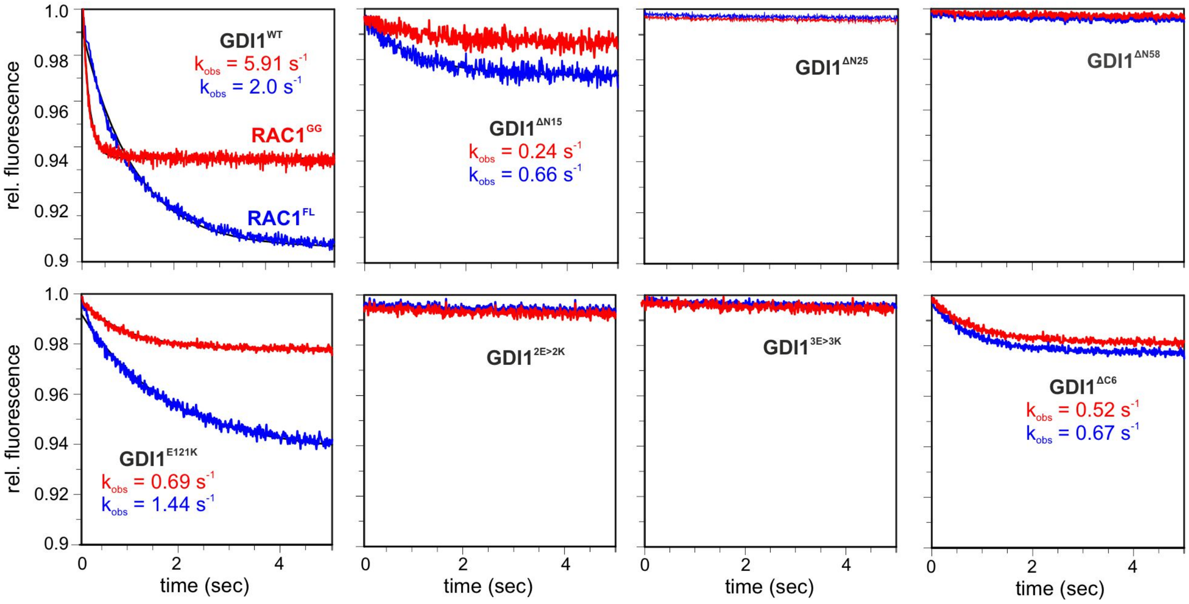

Figure S6. Kinetics of association of GDI1 variants with RAC1^{GG} (red) and RAC1^{FL} (blue). 4 µM GDI1 was rapidly mixed with the indicated RHO GTPases (0.2 µM, respectively) in a stopped-flow apparatus to monitor the association reaction between the two proteins. Observed rate constants (k_{obs}) were calculated by fitting the data to single-exponential decay functions (red line). Evaluated data are shown as bar charts in Figure 3B.

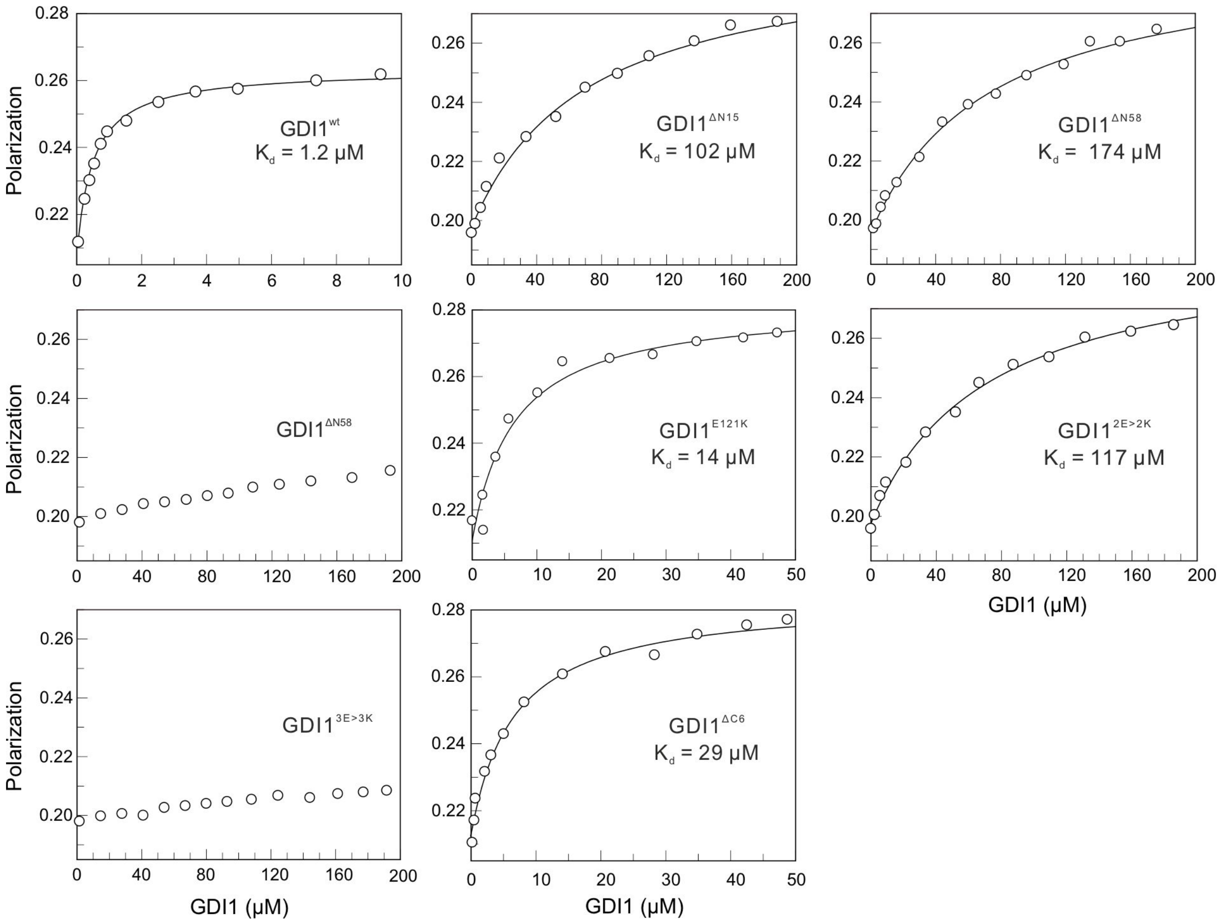

Figure S7. Kinetics of association of GDI1 variants with RAC1^{GG} (red) and RAC1^{FL} (blue). Experiments were performed to determine the dissociation constants (K_d) for the interaction of increasing concentrations of mdGDP-bound RAC1^{WT} with various GDI1 variants as indicated. The X-axis represents the concentration of the SH3 domain as GST fusion proteins in μM and Y-axis represents relative fluorescence polarization. Equilibrium K_d values for the respective interactions, mostly extrapolated due to very low affinities, were calculated by fitting data to a quadratic ligand-binding equation (solid lines). These data are summarized in Figure 4B.

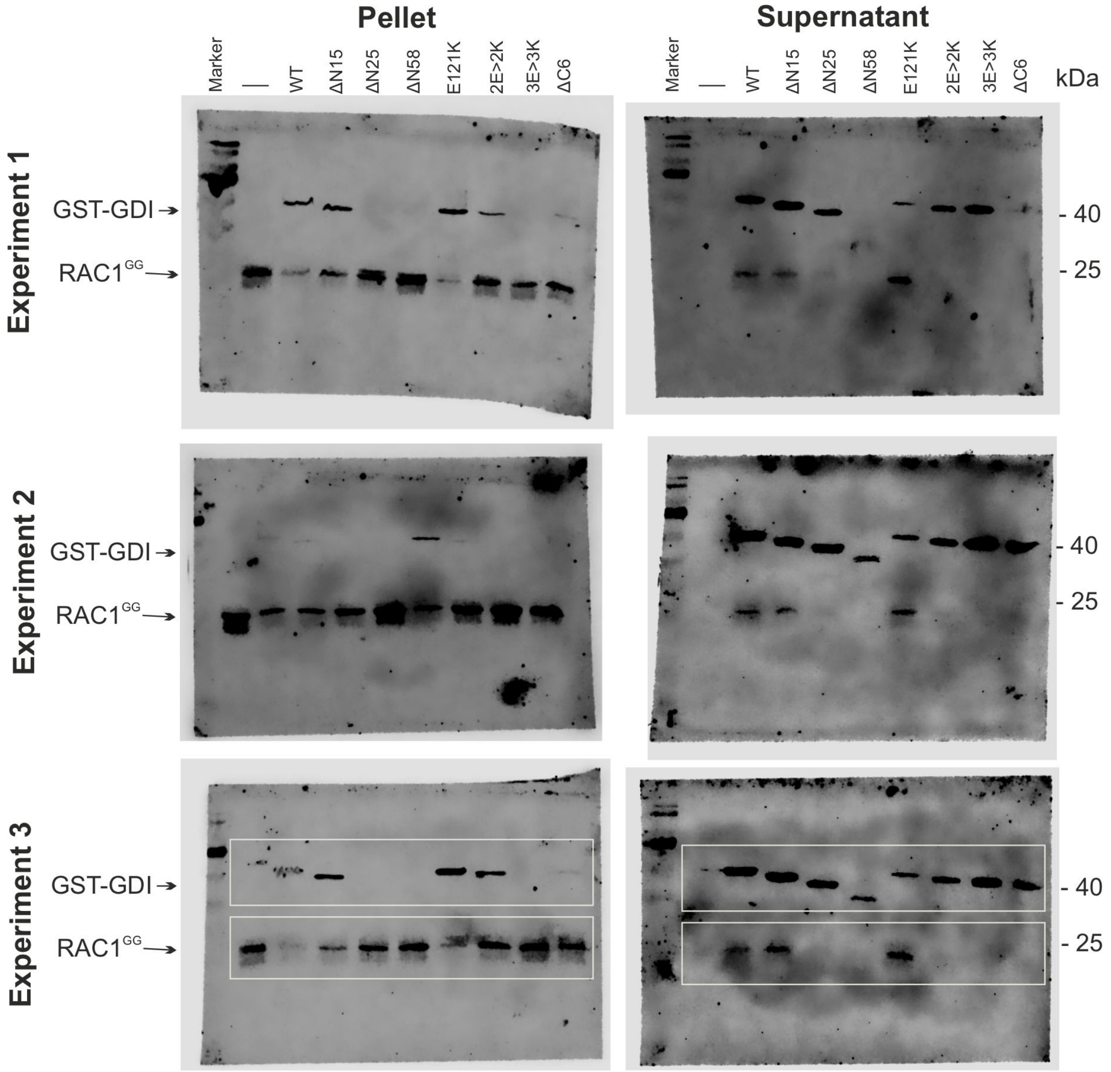

Figure S8. Different GDI construct differentially extract prenylated Rac1 from liposomes. Displacement of GDP-bound RAC1^{GG} from synthetic liposomes by GST-RhoGDI1 variants was studied using liposome sedimentation assay. Briefly, liposomes were generated by using a defined composition of lipids (500 mM) containing 39% w/w phosphatidylethanolamine, 16% w/w phosphatidylcholine, 36% w/w phosphatidylserine, 4% sphingomyelin, and 5 % w/w phosphatidylinositol 4,5-bisphosphate. 3 µg Prenylated RAC1^{GG} was added to the liposomes suspended in protein buffer (20 mM HEPES at pH 7.4, 150 mM NaCl, 5 mM $MgCl_2$, 3 mM DTT) and incubated for 20 min on ice. GST-GDI1 variants (6 µM) were added to the liposome/prenylated Rac1 and further incubated on ice for 30 min. The samples were then centrifuged at 14,000 x g for 30 min at 4 °C. Pellet and supernatant fractions were collected, separated on SDS-PAGE and immunoblotted for RAC1 and GDI1. Bound RAC1 to the liposome was detected in the pellet and extracted RAC1 from liposome via GDI were detected in the supernatant using His tag antibody. The intensity of anti-GST antibody (for GDI1) and anti-His antibody (for RAC1) were detected at an emission wavelength of 700 nm using Odyssey Fc Imaging System (LI-COR Biosciences). White boxes in experiment 3 indicate the blots shown in Figure 4D left panel. Detected signals were quantified using the LI-COR Biosciences Image Studio version 5.2 imaging software. These data are summarized in Figure 4D middle and right panels.

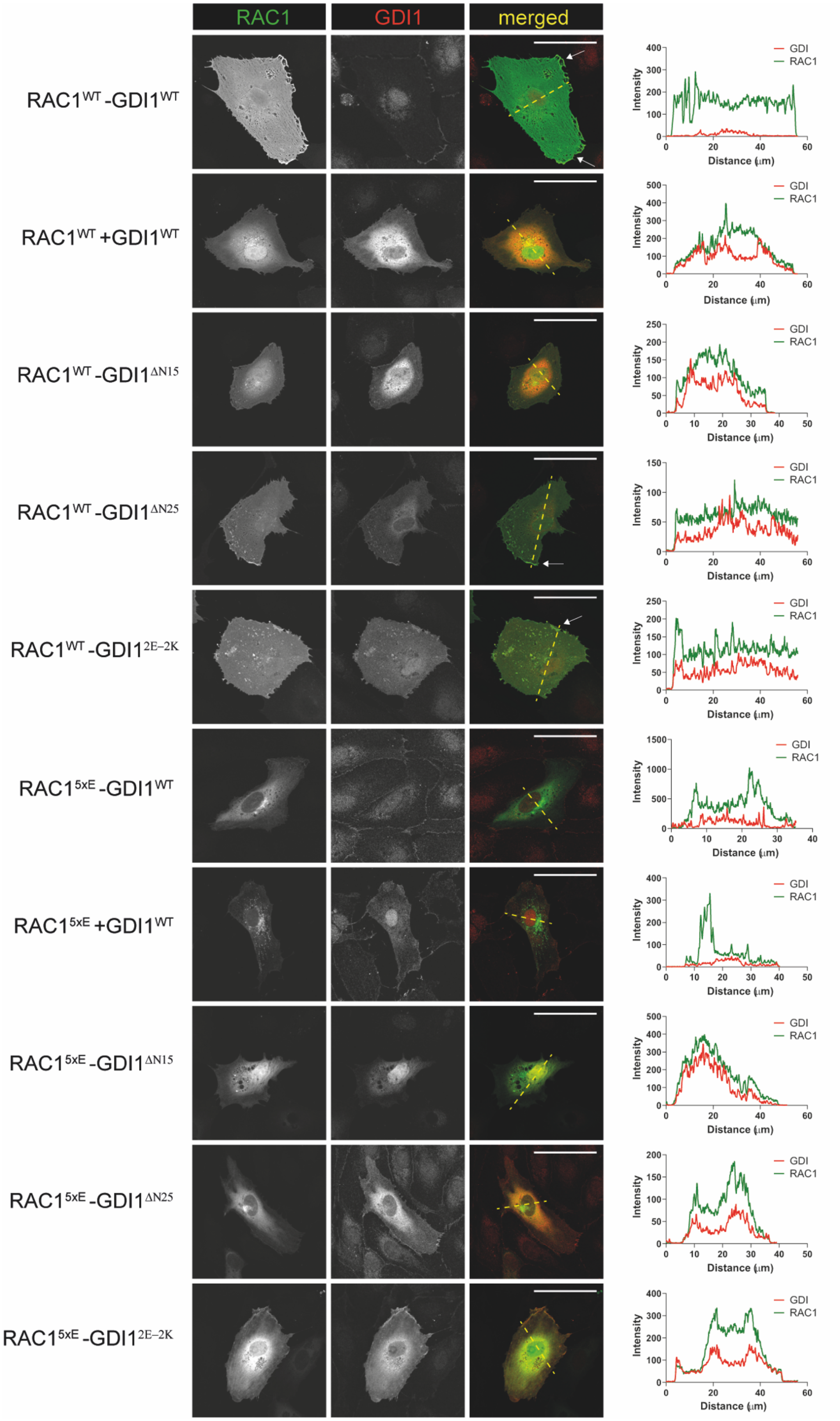
RAC1
GDI1
merged
RAC1WT-GDI1WT
RAC1WT+GDI1WT
RAC1WT-GDI1ΔN15
RAC1WT-GDI1ΔN25
RAC1WT-GDI12E–2K
RAC15xE-GDI1WT
RAC15xE+GDI1WT
RAC15xE-GDI1ΔN15
RAC15xE-GDI1ΔN25
RAC15xE-GDI12E–2K
GDI
RAC1
Intensity
Distance (μm)

Figure S9. Immunofluorescence analysis of YFP-RAC1 and FLAG-GDI1 variants in HUVECs. pYFP-RAC1 and pcDNA3.1-FLAG-GDI1 variants were overexpressed in HUVECs to analyze the molecular basis of their interactions. Single channel images of the RAC1 (green) and GDI1 (red) are shown along with the merged. Scale bar represents 50 μm. Arrows point to colocalization of RAC1 and GDI1 at the plasma membrane. Merged images are shown in Figure 4E (see Materials and Methods for more details). To analyze the distribution of overexpressed RAC1 and GDI1 in the cells, a line was drawn across the cell in ImageJ and graphs showing the fluorescence intensity of RAC1 and GDI1 across the line are shown at the right panel.
